# Environment-Aware Production Scheduling for Paint Shops in Automobile Manufacturing: A Multi-Objective Optimization Approach

**DOI:** 10.3390/ijerph15010032

**Published:** 2017-12-25

**Authors:** Rui Zhang

**Affiliations:** School of Economics and Management, Xiamen University of Technology, Xiamen 361024, China; r.zhang@ymail.com; Tel.: +86-592-6291321

**Keywords:** green manufacturing, automobile industry, pollution reduction, sustainable production scheduling, particle swarm optimization

## Abstract

The traditional way of scheduling production processes often focuses on profit-driven goals (such as cycle time or material cost) while tending to overlook the negative impacts of manufacturing activities on the environment in the form of carbon emissions and other undesirable by-products. To bridge the gap, this paper investigates an environment-aware production scheduling problem that arises from a typical paint shop in the automobile manufacturing industry. In the studied problem, an objective function is defined to minimize the emission of chemical pollutants caused by the cleaning of painting devices which must be performed each time before a color change occurs. Meanwhile, minimization of due date violations in the downstream assembly shop is also considered because the two shops are interrelated and connected by a limited-capacity buffer. First, we have developed a mixed-integer programming formulation to describe this bi-objective optimization problem. Then, to solve problems of practical size, we have proposed a novel multi-objective particle swarm optimization (MOPSO) algorithm characterized by problem-specific improvement strategies. A branch-and-bound algorithm is designed for accurately assessing the most promising solutions. Finally, extensive computational experiments have shown that the proposed MOPSO is able to match the solution quality of an exact solver on small instances and outperform two state-of-the-art multi-objective optimizers in literature on large instances with up to 200 cars.

## 1. Introduction

In recent years, the Chinese government has enforced strict regulations to deal with pollutions in the manufacturing industry [1]. The regulatory pressure urges relevant companies to pay more attention to sustainability aspects of their operational systems with an aim of reducing pollutant emissions. The latest research has revealed that production scheduling could serve as a cost-effective tool for realizing the goal of sustainable manufacturing [2]. For example, Zhang et al. [3] develop a time-indexed integer programming formulation to identify production schedules that minimize energy consumption under TOU (time-of-use) tariffs. Liu and Huang [4] investigate a batch-processing machine scheduling problem and a hybrid flow shop scheduling problem with carbon emission criteria. Zhou et al. [5] apply a genetic algorithm (GA) for the optimization of production schedules in textile dyeing industries with a clear aim of reducing the consumption of fresh water.

Production scheduling is a system-level decision which determines the processing sequence of jobs (orders) in each production unit. Conventional scheduling research has mostly been focused on profit-driven performance indicators such as makespan (for measuring production efficiency) and total flowtime (for measuring work-in-process inventory). To incorporate environmental considerations, it is possible to introduce sustainability-inspired objectives into the scheduling models so that the resulting job processing sequence can achieve a satisfactory trade-off between the traditional performance goal and the pollution reduction goal.

This paper reports a new study based on the motivation and methodology stated above. In particular, we consider the scheduling of a paint shop in automotive manufacturing systems, where pollutant emissions are mainly caused by the frequent cleaning operations performed on painting devices such as spray guns. The cleaning process inevitably leads to discharge of unconsumed paints and detergents which contain hazardous chemicals. Therefore, the scheduling function should attempt to minimize the frequency of cleaning by arranging cars that require identical or similar colors to be processed in a consecutive manner (because a deep cleaning is needed whenever the painting equipment is preparing to switch to a drastically different color for painting). However, considering the requirement on pollution reduction alone is not feasible in practice, due to the fact that the paint shop is coupled with the subsequent assembly shop via a buffer system with limited resequencing capacity, which means the sequencing decision for the paint shop will have a strong impact on possible processing sequences for the assembly shop. In this case, the preferences of the assembly shop must be considered simultaneously and thus should be integrated into the scheduling problem for the paint shop. This clearly defines a bi-objective optimization problem, in which one objective function is concerned with minimization of pollutant emissions while the other objective function reflects the major criterion adopted by the assembly shop (we will consider due date performance in this paper because the assembly shop must strive to deliver finished products on time to the final testing department). To solve such a complicated production scheduling problem with reasonable computational time, we will present a highly modified multi-objective particle swarm optimization (MOPSO) algorithm with enhanced search abilities.

The remainder of this paper is organized as follows. Section 2 provides a brief literature review on the publications related with the scheduling of automotive manufacturing processes. Section 3 introduces the production environment considered in our research (with a focus on the buffer system) and then formulates the studied bi-objective production scheduling problem using a mixed-integer programming model. Section 4 deals with the subproblem of scheduling the assembly shop under a given schedule for the paint shop and the intermediate buffer. Section 5 presents the main algorithm, i.e., the proposed MOPSO for solving the bi-objective integrated production scheduling problem. Section 6 gives the computational results together with statistical tests to compare the proposed algorithm with an exact solver and two high-performing generic multi-objective optimizers published in recent literature. Finally, Section 7 concludes the paper and discusses potential directions for future research.

## 2. Literature Review

### 2.1. The Color-Batching Problem

A line of research that is closely related to our study deals with the color-batching problem, which concerns the use of a buffer system to adjust the car sequence inherited from the upstream body shop so that the resulting sequence best suits the need of the paint shop. In particular, the objective is to minimize the number of color changes (or equivalently, maximizing the average size of color blocks) in the output sequence.

Spieckermann et al. [6] present a formulation of the color-batching process as a sequential ordering problem and propose a branch-and-bound (B&B) algorithm to find the optimal output sequence with the minimum number of color changes. Moon et al. [7] conduct a simulation study for designing and implementing a color rescheduling storage (CRS) in an automotive factory and suggest some simple fill and release policies for operating the selectivity bank. Hartmann and Runkler [8] present two ant colony optimization (ACO) algorithms to enhance simple rule-based color-batching methods. The two ACO algorithms are used for handling the two stages of online resequencing, i.e., filling and releasing, respectively. Sun et al. [9] propose two heuristic procedures (namely, arraying and shuffling heuristics) for achieving quick and effective color-batching. The arraying heuristic is applied in the filling stage, while the shuffling heuristic is used in the releasing stage. Experiments show that the two proposed heuristics can work jointly to obtain competitive color-batching results with very short computational time. Ko et al. [10] investigate the color-batching problem on *M*-to-1 conveyor systems, with motivations from the resequencing problem at a major Korean automotive manufacturer. The authors first develop a mixed-integer linear programming (MILP) model and a dynamic programming (DP) algorithm for a special case of the problem (i.e., 2-to-1 conveyor system), and then propose two efficient genetic algorithms (GAs) to find near-optimal solutions for the general case.

In addition to the above-mentioned method of using a buffer system to resequence cars physically, another way of implementing color-batching is to perform a virtual resequencing of cars. In this case, car positions in the sequence remain unchanged, but the painting colors are reassigned among those cars which share identical product attributes (and therefore are indistinguishable) at the moment. The color-batching problem based on the virtual resequencing strategy is also referred to as the paint shop problem for words (PPW), which has been shown to be NP-complete [11]. Xu and Zhou [12] present four heuristic rules for solving this problem and also propose a beam search (BS) algorithm based on the best heuristic rule. Some researchers, for example Amini et al. [13] and Andres and Hochstättler [14], present heuristic methods for solving a special case of the PPW, i.e., PPW(2,1) or the binary PPW (involving only two painting colors), where each type of car body appears twice in the sequence and have to be painted with different colors. Recently, Sun and Han [15] show that integrated physical and virtual resequencing can generally obtain noticeably better color-batching results than the conventional physical resequencing.

### 2.2. The Car Sequencing Problem

The research on scheduling of automobile manufacturing processes has mainly focused on the car sequencing problem (first described in [16]). The problem concerns the sequencing of cars in the assembly shop, where different options (e.g., sunroof, air-conditioning) are to be installed on the cars by the corresponding workstations distributed along the assembly line. To prevent overload for a workstation, the cars which require the same option have to be spaced out in the processing sequence. Such restrictions are modeled as ratio constraints. Regarding the *r*-th option, the ratio constraint $n_{r}:p_{r}$ indicates that in any subsequence of $p_{r}$ cars, there should be no more than $n_{r}$ cars requiring this option. The scheduling objective is therefore to find a sequence that minimizes the number of constraint violations (NP-hard in the strong sense [17,18]).

Golle et al. [19] present a graph representation of the car sequencing problem and develops an exact solution approach based on iterative beam search. Using improved lower bounds, the proposed approach is shown to be superior to the best known exact solution procedure, and can even be applied to problems of real-world size. In addition to exact solution methods, there are also some approaches built on the hybridization of meta-heuristics and mathematical programming techniques. Zinflou et al. [20] propose three hybrid approaches based on a genetic algorithm which incorporates crossover operators using an integer linear programming model for the construction of offspring solutions. It is shown that the hybrid approaches outperform a genetic algorithm with local search and other algorithms found in the literature. Thiruvady et al. [21] design a hybrid algorithm (also called a matheuristic) by integrating Lagrangian relaxation (LR) and ant colony optimization (ACO) for the car sequencing problem. According to the experiments on various LR heuristics, ACO algorithm, and different hybrids of these methods, it is found that the two-phase LR+ACO method where ACO uses the LR solutions to produce its pheromone matrix is the best-performing method for up to 300 cars.

A very well-known variation of the car sequencing problem is the one proposed by the French car manufacturer Renault and used as subject of the ROADEF’2005 challenge [22]. The Renault problem differs from the standard problem in that, besides ratio constraints imposed by the assembly shop, it also introduces paint batching constraints and priority classifications. The aim is to find a common processing sequence for both the paint shop and the assembly shop such that a lexicographically defined objective function is minimized. Due to the large number of cars and tight computational time limit, the algorithms that rank the first 10 places in the final competition all belong to the heuristic category. Estellon et al. [23] describe a first-improvement descent heuristic using a variety of neighborhood operators randomly, which is further enhanced by a strategy to speed up neighborhood evaluations through the use of incremental calculation. Ribeiro et al. [24] design a set of heuristics mostly based on variable neighborhood search (VNS) and iterated local search (ILS). Quick neighborhood evaluations and ad-hoc data structures are also a key feature in their method. Briant et al. [25] present a simulated annealing (SA) algorithm in which the probabilities of acceptance are computed dynamically so that the search process tends to favor the moves that have the best success rate so far among all the possible moves. Gavranović [26] presents a heuristic based on variable neighborhood search (VNS) and tabu search (TS). The author also proposes a data structure to speed up penalty evaluation for ratio constraints and exploits the concept of an alphabet to improve the number of batch colors. As a matter of fact, the Renault version of car sequencing problem has attracted long-lasting research interest. Most recently, Jahren and Achá [27] revisit this problem to investigate how to close the gap between exact and heuristic methods. The authors report new lower bounds for 7 out of the 19 instances used in the final round of the competition by applying an improved integer programming formulation. In addition, a novel column generation-based exact algorithm is proposed for solving the problem, which outperforms an existing branch-and-bound approach.

Besides the standard version and the Renault version of car sequencing problems, researchers are also studying extended car sequencing problems with additional constraints, objective functions or decision types. Zhang et al. [28] propose an artificial ecological niche optimization (AENO) algorithm for a car sequencing problem with an additional objective of minimizing energy consumption in the sorting process, which is an important issue but often neglected in previous research. Computational experiments show that the proposed AENO algorithm achieves competitive results compared with the most effective heuristics for the conventional objectives, and in the meantime, it realizes considerable reduction of energy consumption. Yavuz [29] studies the combined car sequencing and level scheduling problem which aims at finding the optimal production schedule that evenly distributes different models over the planning horizon and meanwhile satisfies all ratio constraints for the options. The author proposes a parametric iterated beam search algorithm for the combined scheduling problem, which can be used either as a heuristic or as an exact solution method. Chutima and Olarnviwatchai [30] apply a special version of the estimation of distribution algorithm (EDA) called extended coincident algorithm (COIN-E) to a multi-objective car sequencing problem based on a more realistic production setting, namely, two-sided assembly lines. Three objectives are minimized simultaneously in the Pareto sense, including the number of paint color changes, the number of ratio constraint violations and the utility work (i.e., uncompleted operations which must be finished by additional utility workers).

Based on the literature review, the limitations of previous research can be summarized in the following two aspects. Firstly, the existing scheduling models require that the same processing sequence is adopted by both the paint shop and the assembly shop, without considering the resequencing opportunity provided by a buffer connecting the two shops. Secondly, the ratio constraints for installing options in the assembly shop have been overemphasized while the environmental impact of the cleaning operations in the paint shop have not been precisely measured. In fact, most of the modern automobile manufacturers adopt a buffer system to connect the shops, and the increased level of automation suggests that the ratio constraints are not so binding as before. On the other side, environmental protection has evidently become a major concern in the manufacturing sector. Under such a background, this paper aims at dealing with the new challenge on sustainability-conscious production scheduling in the contemporary car manufacturing industry.

## 3. Problem Formulation

### 3.1. The Production Setting

**Figure 1:**
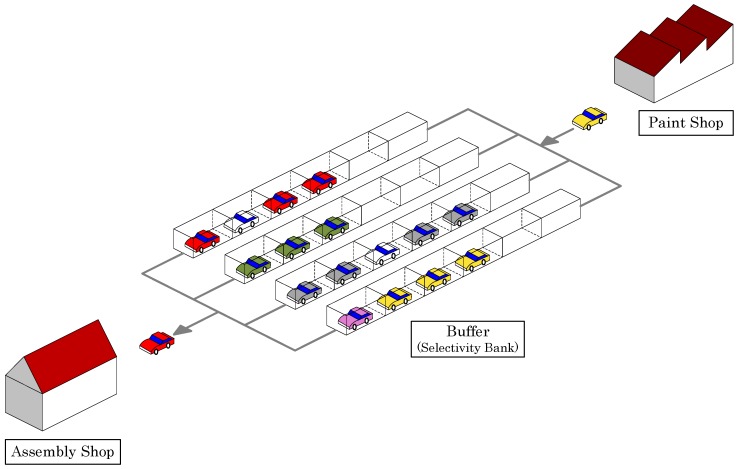
Illustration of a production system for automotive painting and assembly.

In a typical automotive manufacturing system, painting and assembly operations are performed in two sequential workshops connected with a resequencing buffer. Figure 1 provides an illustration of the production setting considered in this study.

Suppose that a set of *n* cars {1,2,…,n} are to be processed successively in the paint shop. When the next car in the processing sequence requires a different color than the previous car, a setup operation is needed to clean the painting equipment (e.g., spray guns) thoroughly. The cleaning procedure is accompanied by the use of a chemical detergent, and the resulting discharge of unconsumed paint will directly lead to sewage emissions. Therefore, it is desirable to have the cars with identical or similar colors processed consecutively (as blocks) so as to reduce the frequency of color changes in the processing sequence of paint shop. Formally, for any two colors ($e_{1}$ and $e_{2}$) from the set of all possible colors {1,2,…,E}, let $\delta_{e_{1}e_{2}}$ denote the amount of pollutant emissions caused by the setup operation that is needed between the painting of $e_{1}$ and the painting of $e_{2}$. For example, when a light color immediately follows a dark color, the painting devices need a deep cleaning and consequently the emission level is higher. The aim of paint shop scheduling is to minimize the total amount of pollutant emissions produced by the cleaning operations.

Once the cars have been painted, they will be released from the paint shop one after another in their original order. Then, there will be an opportunity to resequence the cars by utilizing the buffer system in order to meet the preferences of the subsequent assembly shop. In this study, we consider the due date preferences. Formally, for each car *i*, a due date $d_{i}$ is given according to the requirement of customers, which is expressed in terms of latest position in the production sequence. For example, if car *i* is preferred to be sequenced in the first 10 positions in the assembly shop, we set $d_{i}=10$, which means a tardiness cost will be incurred in case the car is placed after the 10th position in the processing sequence. Position-based due date specification makes sense because the production in assembly shop is organized according to a fixed cycle time (also known as paced assembly line). Once the processing sequence is fixed, the time of completing and releasing each car from the assembly shop is also known. In addition, a priority weight $w_{i}$ is assigned to each car *i*, which reflects the relative value and importance of its related customer. The aim of assembly shop scheduling is therefore to minimize the total weighted tardiness defined as $\sum_{i=1}^{n}w_{i}{\left({\hat{\pi }}_{i}-d_{i}\right)}^{+}$, where ${\left(x\right)}^{+}=\max\left\{x,0\right\}$ and ${\hat{\pi }}_{i}$ represents the actual position of car *i* in the processing sequence of assembly shop. In this study, we have ignored the ratio constraints (cf. Section 2.2) based on the following two observations (particularly for Chinese car manufacturers). First, the advanced automation technology applied in contemporary car assembly lines significantly reduces the occurrence of workstation overloading. Second, manufacturing of medium-grade cars has gradually transformed to a mass production mode, which means the number of independent optional features has decreased. Despite these facts, however, it must be noted that ratio constraints are still important concerns for European (and more specifically German) manufacturers of high-end cars.

According to the above descriptions, it is very clear that paint shop and assembly shop have their individual goals which are in fact mutually independent. The major difficulty in the integrated scheduling of both shops arises from the fact that the resequencing buffer connecting them has a limited capacity, which means the *n* cars cannot be completely resequenced after leaving the paint shop and before entering the assembly shop. Therefore, it is necessary to make a compromise between the goal of paint shop (total pollutant emissions) and the goal of assembly shop (total weighted tardiness) by building a bi-objective optimization model that is able to produce a set of Pareto solutions for the decision-makers.

### 3.2. The Resequencing Buffer System

The buffer system that connects paint shop and assembly shop offers an opportunity to partially resequence the cars. Among the various types of buffer systems mentioned in [31], selectivity bank (also referred to as mix bank) is the most commonly used system for physical resequencing in the automobile manufacturing industry because of its low cost and high flexibility.

A selectivity bank consists of *L* parallel lanes, where the *l*-th lane has $c_{l}$ spaces for storing cars. At the entrance of the buffer, a car may choose to enter any of the lanes with unoccupied spaces and join the queue in that lane. At the exit of the buffer, the first car in any nonempty lane may be released into the processing sequence for assembly shop. Clearly, the resequencing ability of a selectivity bank depends on the number of lanes. If L=n, then it can realize a complete resequencing of *n* cars and output any sequence as needed. In reality, however, *L* is certainly much less than the number of cars to be resequenced, and therefore the selectivity bank can only implement a partial resequencing of cars. The general rule is: it is not possible to change the relative order of two cars if they have entered the same lane (because each lane is equivalent to a first-in first-out queue structure).

**Figure 2:**
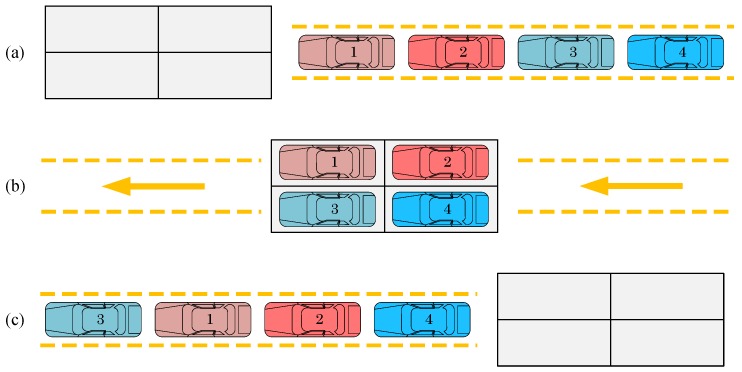
An example of selectivity bank with two lanes.

Figure 2 gives an example to illustrate the function of a selectivity bank with two lanes (L=2) and two spaces in each lane ($c_{1}=c_{2}=2$). Initially, the four cars are sequenced according to their indexes, i.e., in the order of 1, 2, 3, 4 (Step (a)). To move car 3 to the very first position, we must let car 3 enter a different lane than the one chosen by car 1 and car 2 because car 3 needs to “jump” over the two cars (Step (b)). Finally, car 3 is first released from the selectivity bank, and consequently the sequencing of the four cars is altered to 3, 1, 2, 4 (Step (c)). Note that it is not possible to realize a sequence like 3, 2, 1, 4 because of the limited number of lanes.

### 3.3. The MILP Model

We will formulate the paint shop scheduling problem as a mixed-integer linear programming (MILP) model. First, a group of 0−1 decision variables are introduced as follows.

(1) $$\begin{array}{cc} x_{ik} & =\left\{\begin{array}{cc} 1 & \mathrm{if}\;\mathrm{car}\;i\;\mathrm{is}\;\mathrm{processed}\;\mathrm{in}\;\mathrm{the}\;k-\mathrm{th}\;\mathrm{position}\;\mathrm{in}\;\mathrm{paint}\;\mathrm{shop},\\ 0 & \mathrm{otherwise}. \end{array}\right. \end{array}$$

(2) $$\begin{array}{cc} {\hat{x}}_{ik} & =\left\{\begin{array}{cc} 1 & \mathrm{if}\;\mathrm{car}\;i\;\mathrm{is}\;\mathrm{processed}\;\mathrm{in}\;\mathrm{the}\;k-\mathrm{th}\;\mathrm{position}\;\mathrm{in}\;\mathrm{assembly}\;\mathrm{shop},\\ 0 & \mathrm{otherwise}. \end{array}\right. \end{array}$$

(3) $$\begin{array}{cc} y_{ek} & =\left\{\begin{array}{cc} 1 & \mathrm{if}\;\mathrm{the}\;\mathrm{car}\;\mathrm{processed}\;\mathrm{in}\;\mathrm{the}\;k-\mathrm{th}\;\mathrm{position}\;\mathrm{in}\;\mathrm{paint}\;\mathrm{shop}\;\mathrm{requires}\;\mathrm{color}\;e,\\ 0 & \mathrm{otherwise}. \end{array}\right. \end{array}$$

(4) $$\begin{array}{cc} z_{il} & =\left\{\begin{array}{cc} 1 & \mathrm{if}\;\mathrm{car}\;i\;\mathrm{enters}\;\mathrm{the}\;l-\mathrm{th}\;\mathrm{lane}\;\mathrm{in}\;\mathrm{the}\;\mathrm{buffer}\;\mathrm{area},\\ 0 & \mathrm{otherwise}. \end{array}\right. \end{array}$$

With these decision variables, the complete MILP model can be established. Note that the other decision variables in the following model (e.g., $Y_{e_{1}e_{2}k}$, $\Phi_{ij}$, $T_{i}$) are all defined on the basis of these fundamental variables. We use *M* to denote a very large positive number.

(5) $$\begin{array}{cc} \mathrm{Minimize}\; & TPE=\sum_{k=2}^{n}\sum_{e_{1}=1}^{E}\sum_{e_{2}=1}^{E}\left(\delta_{e_{1}e_{2}}\cdot Y_{e_{1}e_{2}k}\right) \end{array}$$

(6) $$\begin{array}{c} TWT=\sum_{i=1}^{n}\left(w_{i}\cdot T_{i}\right) \end{array}$$

(7) $$\begin{array}{cc} \mathrm{subject}\;\mathrm{to}\text{:}\; & \sum_{i=1}^{n}x_{ik}=\sum_{i=1}^{n}{\hat{x}}_{ik}=1,\;k=1,\ldots ,n \end{array}$$

(8) $$\begin{array}{c} \sum_{k=1}^{n}x_{ik}=\sum_{k=1}^{n}{\hat{x}}_{ik}=1,\;i=1,\ldots ,n \end{array}$$

(9) $$\begin{array}{c} \sum_{l=1}^{L}z_{il}=1,\;i=1,\ldots ,n \end{array}$$

(10) $$\begin{array}{c} \sum_{i=1}^{n}z_{il}\leq c_{l},\;l=1,\ldots ,L \end{array}$$

(11) $$\begin{array}{c} y_{ek}=\sum_{i=1}^{n}\left(\beta_{ei}\cdot x_{ik}\right),\;k=1,\ldots ,n,\;e=1,\ldots ,E \end{array}$$

(12) $$\begin{array}{c} Y_{e_{1}e_{2}k}\geq y_{e_{1}\left(k-1\right)}+y_{e_{2}k}-1,\;k=1,\ldots ,n,\;e_{1},e_{2}=1,\ldots ,E \end{array}$$

(13) $$\begin{array}{c} Y_{e_{1}e_{2}k}\geq 0,\;k=1,\ldots ,n,\;e_{1},e_{2}=1,\ldots ,E \end{array}$$

(14) $$\begin{array}{c} \sum_{k=1}^{n}\left(k\cdot x_{ik}\right)-\sum_{k=1}^{n}\left(k\cdot x_{jk}\right)\leq M\cdot \Phi_{ij},\;i,j=1,\ldots ,n \end{array}$$

(15) $$\begin{array}{c} \sum_{k=1}^{n}\left(k\cdot x_{ik}\right)-\sum_{k=1}^{n}\left(k\cdot x_{jk}\right)\geq M\cdot \left(\Phi_{ij}-1\right),\;i,j=1,\ldots ,n \end{array}$$

(16) $$\begin{array}{c} \sum_{k=1}^{n}\left(k\cdot {\hat{x}}_{ik}\right)-\sum_{k=1}^{n}\left(k\cdot {\hat{x}}_{jk}\right)\leq M\cdot \left(2-z_{il}-z_{jl}\right)+M\cdot \Phi_{ij},\;i,j=1,\ldots ,n,\;l=1,\ldots ,L \end{array}$$

(17) $$\begin{array}{c} T_{i}\geq \sum_{k=1}^{n}\left(k\cdot {\hat{x}}_{ik}\right)-d_{i},\;i=1,\ldots ,n \end{array}$$

(18) $$\begin{array}{c} T_{i}\geq 0,\;i=1,\ldots ,n \end{array}$$

(19) $$\begin{array}{c} x_{ik},{\hat{x}}_{ik},y_{ek},z_{il},\Phi_{ij}\in \left\{0,1\right\},\;i,k=1,\ldots ,n,\;e=1,\ldots ,E,\;l=1,\ldots ,L \end{array}$$

Equation (5) defines the first objective, i.e., minimizing the total pollutant emissions (TPE) caused by the setup operations in paint shop ($\delta_{e_{1}e_{2}}$ represents the amount of emissions during a setup operation to switch from color $e_{1}$ to color $e_{2}$). The binary variable $Y_{e_{1}e_{2}k}$ is defined in such a way that $Y_{e_{1}e_{2}k}=1$ if and only if the car in the (k−1)-th position is painted with color $e_{1}$ (i.e., $y_{e_{1}\left(k-1\right)}=1$) and meanwhile the car in the *k*-th position is painted with color $e_{2}$ (i.e., $y_{e_{2}k}=1$); $Y_{e_{1}e_{2}k}=0$ otherwise (i.e., either $y_{e_{1}\left(k-1\right)}=0$ or $y_{e_{2}k}=0$). Equation (6) defines the second objective, i.e., minimizing the total weighted tardiness (TWT) incurred by late finishing of cars in the subsequent assembly shop ($w_{i}$ represents the priority weight of car *i*). Equations (7) and (8) reflect the assignment constraints, i.e., each car should be assigned to exactly one position in the processing sequence and each position in the sequence must be occupied by exactly one car. Likewise, Equation (9) specifies that each car can only choose to enter one lane of the selectivity bank. Equation (10) requires that the number of cars entering lane *l* should not exceed its capacity denoted by $c_{l}$ (we can assume $c_{l}=\infty$ if the cars move through the buffer in a dynamic manner). Equation (11) defines $y_{ek}$, which equals 1 if and only if the car in the *k*-th position should be painted with color *e*. Note that $\beta_{ei}$ is a parameter known in advance ($\beta_{ei}=1$ if car *i* requires color *e* and $\beta_{ei}=0$ otherwise). Equations (12) and (13) provide the definition for $Y_{e_{1}e_{2}k}$ based on $y_{e_{1}\left(k-1\right)}$ and $y_{e_{2}k}$ (note that the two inequalities are enough to make $Y_{e_{1}e_{2}k}$ binary variables). Equations (14) and (15) are used to define $\Phi_{ij}$, which depicts the relative order of two cars *i* and *j* in the paint shop: $\Phi_{ij}=1$ if car *i* is processed after car *j*, and $\Phi_{ij}=0$ if car *i* is processed before car *j*. We need $\Phi_{ij}$ as intermediate variables to reflect the impact of the paint shop sequence on the possible sequences for assembly shop (note that $\Phi_{ij}$ is used in the following Equation (16)). Equation (16) describes the constraint imposed by the selectivity bank: the relative order of two cars cannot be altered if they travel through the same lane. In particular, if car *i* has been scheduled before car *j* in the paint shop (i.e., $\Phi_{ij}=0$) and both cars have entered the *l*-th lane of the buffer (i.e., $z_{il}+z_{jl}=2$), then car *i* is definitely positioned before car *j* when they leave the buffer for the next production step. Equations (17) and (18) evaluate the tardiness of car *i* in the assembly shop, which is defined as $T_{i}=\max\left\{{\hat{\pi }}_{i}-d_{i},0\right\}$, with ${\hat{\pi }}_{i}$ denoting the position of car *i* in the processing sequence of assembly shop and $d_{i}$ representing the preferred latest position of car *i*.

### 3.4. Further Discussion

The studied production system consists of two sequential stages with an additional intermediate buffer. Minimizing the TPE in the first production stage is equivalent to minimizing the sum of sequence-dependent setup times (more accurately, it is the same as the problem $1|s_{ij}|C_{\max}$[32] which is further equivalent to the traveling salesman problem and thus strongly NP-hard). The problem of the second stage is minimizing TWT under precedence constraints (more precisely, $1|\mathrm{chains};p_{j}=1|\sum w_{j}T_{j}$), which is also NP-hard in the strong sense (more details are given in Section 4).

In fact, our problem is much more complicated than the two subproblems mentioned above because of the resequencing buffer located between the two production stages. The buffer is used for partially resequencing the cars after they leave the first stage and before they enter the second stage. In other words, the two stages can process different car sequences. However, the production system does not fall under the category of flow shops because it is impossible to generate an arbitrary processing sequence for the second stage given the processing sequence in the first stage (the buffer has a limited number of lanes and consequently it can only realize partial resequencing of the cars). What complicates the problem is the fact that the buffer system also requires an optimized decision regarding the allocation of cars to each lane.

Now it is fairly clear that scheduling decisions for the two production stages are tightly coupled. Solving the first-stage problem to optimality may lead to poor performance in terms of the second-stage criterion, and vice versa, which means the strategy of solving each subproblem individually for each production stage is apparently infeasible for addressing the whole problem. The only way of resolving the problem is to build an integrated scheduling model by incorporating the constraints imposed by the resequencing buffer. In this way, it is possible to take the preferences of both stages into consideration and obtain well-balanced schedules for the entire production system. This is exactly the motivation behind the integrated problem formulation.

## 4. The Assembly Shop Sequencing Subproblem

The main optimization algorithm which will be detailed in Section 5 deals with the decisions on car sequencing in the paint shop as well as the allocation of cars to the different lanes of the selectivity bank. A critical issue that arises in the meantime is how to sequence the cars in the downstream assembly shop under a fixed placement of cars in the selectivity bank. This subproblem needs to be solved properly for the evaluation of solutions in the main algorithm.

The assembly shop sequencing subproblem can be described as $1|\mathrm{chains};p_{j}=1|\sum w_{j}T_{j}$ according to the three-field notation system, based on the following observations:

- The paced production mode in assembly shop means that each job has identical processing time ($p_{j}=p$). In addition, the position-based due date assignment scheme suggests that the situation can be further simplified as $p_{j}=1$.
- Each lane of the selectivity bank is actually imposing a set of precedence constraints (in the chain form) on the relevant cars traveling through the lane. These precedence constraints must be respected when scheduling the assembly shop.

**Lemma** **1.** The problem $1|p_{j}=1|\sum w_{j}T_{j}$ is polynomially solvable.

**Proof.**  It can be shown that this problem is equivalent to the Assignment Problem (concerning the assignment of *n* jobs to *n* consecutive positions on a single machine):

(20) $$\begin{array}{cc} \min\; & \sum_{i=1}^{n}{j=1}^{n}C\left(i,j\right)x_{ij} \end{array}$$

(21) $$\begin{array}{cc} s.t.\; & \sum_{j=1}^{n}x_{ij}=1,\;i=1,\ldots ,n \end{array}$$

(22) $$\begin{array}{c} \sum_{i=1}^{n}x_{ij}=1,\;j=1,\ldots ,n \end{array}$$

(23) $$\begin{array}{c} x_{ij}\in \left\{0,1\right\},\;i=1,\ldots ,n,\;j=1,\ldots ,n \end{array}$$

The equivalence is established by setting the cost of assigning job *i* to the *j*-th position as $C\left(i,j\right)=w_{i}\cdot \max\left\{j-d_{i},0\right\}$. Therefore, the Hungarian algorithm can solve this problem within $O(n^{3})$ time. ☐

**Lemma** **2.** The problem $1|\mathrm{chains};\;p_{j}=1|\sum w_{j}T_{j}$ is NP-hard in the strong sense.

For proof of the lemma, readers are suggested to refer to the work of Leung and Young [33].

### 4.1. A Branch-and-Bound Algorithm

In view of the complexity results presented above, we propose a branch-and-bound (B&B) algorithm to solve the problem $1|\mathrm{chains};p_{j}=1|\sum w_{j}T_{j}$, using solutions of the relaxed problem $1|p_{j}=1|\sum w_{j}T_{j}$ as the basis for bounding. Schedules are constructed from the end to the front, i.e., backwards in time, considering the fact that the larger values of weighted tardiness are likely to correspond to the jobs that are scheduled more towards the end of the processing sequence. Therefore, it appears to be beneficial to schedule these jobs first in the branch-and-bound procedure. At the *q*-th level of the search tree, jobs are selected for the (n−q+1)-th position. Under the *L* sets of chain-based precedence constraints, there are at most *L* branches going from each node at level *q* to level q+1 (because only the last unscheduled job in each chain may be considered). It follows that the number of nodes at level *q* is bounded by $L^{q}$. The solution of the relaxed problem without precedence constraints provides a lower bound for the original problem $1|\mathrm{chains};p_{j}=1|\sum w_{j}T_{j}$. This bounding strategy is applied to the set of unscheduled jobs at each node of the search tree. If the lower bound (LB) is larger than or equal to the objective value of any feasible schedule, then the node will be discarded. The complete algorithm is formally described as Algorithm 1.

In the following, we make some comments for better explaining the algorithm. In Line 1, the variable for recording the best objective value obtained so far ($TWT_{\min}$) is initialized to be a very large positive number, and the set of nodes (N) is initialized with the root node which corresponds to a null sequence $N^{0}$ (the job to be put in each position is pending). The lower bound for $N^{0}$ is unimportant and thus $LB(N^{0})$ is assigned with 0. The tree-type search is then started and the search process will be continued until the node set becomes empty. In each iteration, three major steps are performed, i.e., node selection, branching, and handling of offspring nodes.

1. The algorithm always selects the node with the smallest lower bound from N for further exploration (Line 3). The motivation is to focus on the promising subregion of the search space so that it is likely to discover a feasible solution with lower objective value, leading to more opportunities of pruning the search tree.
2. If the selected node $N^{c}$ has a lower bound below the current best objective value (upper bound), the algorithm has to further explore the node by creating branches on it. This is implemented in Line 6. In particular, the algorithm is attempting to insert jobs into the last vacant position of the current partial solution corresponding to $N^{c}$. Constrained by the precedence relations given in the form of $P_{1},\ldots ,P_{L}$, only the last unscheduled job in each precedence chain $P_{l}$ (l=1,…,L) is applicable for this purpose. Hence, at most *L* descendant nodes will be created.
3. For each descendant node $N_{l}^{c}$, the lower bound $LB(N_{l}^{c})$ is first obtained by employing a Hungarian algorithm to solve the relaxed scheduling problem (neglecting precedence constraints) which consists of the unscheduled jobs with respect to the partial solution of $N_{l}^{c}$ (Line 8). Then, three cases are identified and handled separately.
  - If the schedule obtained after solving the relaxed problem ($\pi_{l}^{c}$) turns out to be a feasible solution for the original problem (which means respecting all the precedence relations), then the algorithm further investigates whether this solution defines a new upper bound and updates the relevant variables when necessary (Lines 11–14).
  - If the obtained schedule is not feasible for the original problem but its objective value (i.e., the lower bound $LB(N_{l}^{c})$) turns out to be larger than or equal to the current upper bound (i.e., $TWT_{\min}$), the node $N_{l}^{c}$ can be discarded or fathomed (Line 16) because there is no hope of finding better solutions by branching on $N_{l}^{c}$.
  - Finally, if the lower bound value is below the upper bound, the node should be explored in the subsequent search process, and therefore, it is added to the node set (Line 18).

**Algorithm 1** B&B for $1|\mathrm{chains};p_{j}=1|\sum w_{j}T_{j}$**Input:** The weight ($w_{j}$) and due date ($d_{j}$) of each job j=1,…,n, and *L* chains of jobs indicating their precedence relations ($P_{1},\ldots ,P_{L}$)1:Let $TWT_{\min}=\infty$ and the node set $N=\{\left(N^{0},LB\left(N^{0}\right)\right)\}$, where $N^{0}=\left(*,\ldots ,*\right)$ is an empty sequence of *n* positions, with $LB(N^{0})=0$;2:**while** N≠∅ **do**3: Select the node $N^{c}$ with minimum LB from N (i.e., to satisfy $LB\left(N^{c}\right)=\min_{N\in N}LB\left(N\right)$);4: $N\leftarrow N\;\backslash \{N^{c}\}$;5: **if** $LB\left(N^{c}\right)<TWT_{\min}$ **then**6:  Branch on $N^{c}$: locate the last unscheduled job (if any) in each chain $P_{1},\ldots ,P_{L}$, and put it into the last empty position in $N^{c}$, thereby generating *L* nodes ($N_{1}^{c},\ldots ,N_{L}^{c}$);7:  **for** l=1 *L* **do**8:   Bound $N_{l}^{c}$: evaluate the lower bound $LB(N_{l}^{c})$ by solving the relaxed problem $1|p_{j}=1|\sum w_{j}T_{j}$ for the unscheduled jobs (the schedule obtained is denoted as $\pi_{l}^{c}$);9:   **if** $\pi_{l}^{c}$ is a feasible solution with respect to $P_{1},\ldots ,P_{L}$ **then**10:    Let $TWT\left(\pi_{l}^{c}\right)=LB\left(N_{l}^{c}\right)$;11:    **if** $TWT\left(\pi_{l}^{c}\right)<TWT_{\min}$ **then**12:     $TWT_{\min}\leftarrow TWT\left(\pi_{l}^{c}\right)$;13:     $\pi_{\mathrm{opt}}\leftarrow \pi_{l}^{c}$;14:    **end if**15:   **else if** $LB\left(N_{l}^{c}\right)\geq TWT_{\min}$ **then**16:    Discard $N_{l}^{c}$ (i.e., node $N_{l}^{c}$ is fathomed);17:   **else**18:    $N\leftarrow N\cup \{\left(N_{l}^{c},LB\left(N_{l}^{c}\right)\right)\}$;19:   **end if**20:  **end for**21: **end if**22:**end while****Output:** The optimal solution $\pi_{\mathrm{opt}}$ and its objective value $TWT(\pi_{\mathrm{opt}})$

### 4.2. An Illustrative Example

Consider a small instance with 4 jobs, the details of which are shown in Table 1. The precedence constraints are given by two chains $P_{1}=\left(1\to 4\right)$ and $P_{2}=\left(2\to 3\right)$, which means job 1 must precede job 4 and job 2 must precede job 3.

**Figure 3:**
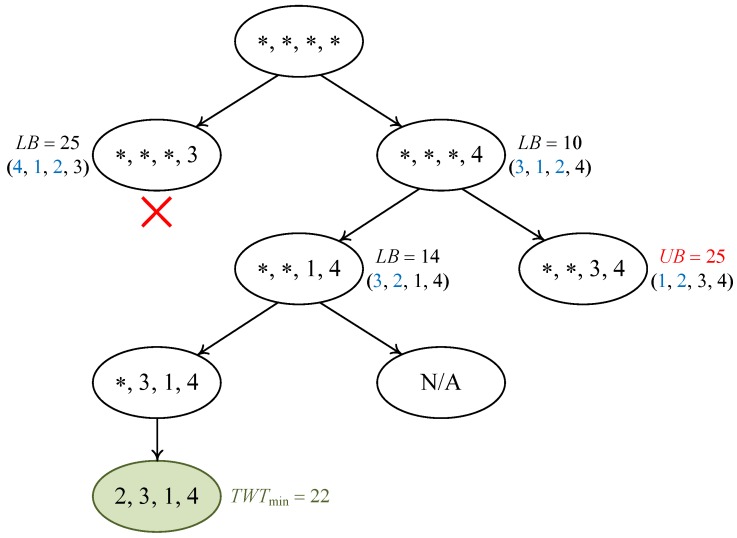
Example search process of the branch-and-bound algorithm.

The process of solving this instance with the proposed B&B algorithm is illustrated in Figure 3. The search begins with a null sequence which corresponds to the root node of the tree. In the first iteration, we branch on this node by determining the job for the last position. According to the precedence chains, only job 3 and job 4 are eligible, and thus two offspring nodes are created on level 1. Solving the relaxed subproblem for the left-hand node (for jobs 1, 2 and 4), we obtain the optimal sequence (4,1,2) with objective value 25. This is not a feasible solution because job 4 is positioned before job 1, which violates the precedence chain $P_{1}$. So the left-hand node is associated with a lower bound of 25. Similarly, for the right-hand node, we obtain a lower bound of 10. In the second iteration, we pick out the node (∗,∗,∗,4), because it has a smaller LB, and then create branches on it by choosing the job for the penultimate position. Since job 4 has been scheduled, the last unscheduled job in each chain is recognized to be job 1 (according to $P_{1}$) and job 3 (according to $P_{2}$), respectively. Thus, two new nodes are produced on level 2, where the left-hand node leads to a lower bound value of 14 while the right-hand node results in a feasible solution (1,2,3,4) (satisfying both $P_{1}$ and $P_{2}$) with objective value 25. In this case, the upper bound $TWT_{\min}$ is updated to 25 and consequently the node (∗,∗,∗,3) is eliminated because $LB\left(*,*,*,3\right)\geq TWT_{\min}$. In the third iteration, we branch on the only node left in the active node set, i.e., (∗,∗,1,4), and create one descendant node on level 3, considering that there is currently no unscheduled job in the chain $P_{1}$ and only job 3 in the chain $P_{2}$ is applicable for the second position. This new node directly leads to a feasible solution (2,3,1,4) with objective value 22, which turns out to be the optimal solution to the problem.

## 5. The Main Algorithm: MOPSO

### 5.1. Fundamentals of PSO

To start with, we provide a brief introduction of the basic principles of standard particle swarm optimization (PSO), which serves as the foundation for our multi-objective PSO algorithm.

The PSO algorithm mimics the flocking behavior of birds in the search of optimal solutions for single-objective continuous function optimization [34]. PSO starts with an initial population of particles (scattered randomly over the search space), which represent potential solutions to the considered problem. Each particle is associated with a fitness value which is obtained by evaluation of the objective function. A velocity vector is used to control the flying direction and speed of each particle. In each iteration, the velocity of each particle gets updated based on a trade-off between two incentives: (1) continuing its current flying direction (i.e., inertia); (2) aiming for the best-so-far positions that it knows (i.e., learning). In particular, there are two types of best-so-far positions. One type is the best position that each particle *i* has visited in its own search history, which is named the personal best position ($pbest_{i}$). The other type is the best position discovered so far by all particles in the swarm, which is named the global best position (gbest). The PSO algorithm iteratively updates the velocity and position for each particle until the convergence condition is satisfied.

Formally, let $x_{i}=\left(x_{i,1},x_{i,2},\ldots ,x_{i,D}\right)$ and $v_{i}=\left(v_{i,1},v_{i,2},\ldots ,v_{i,D}\right)$ respectively denote the position and the velocity of the *i*-th particle (i=1,2,…,N) in a *D*-dimensional search space. The personal best position of particle *i*, which records the best solution found by this particle, is denoted by $b_{i}=\left(b_{i,1},b_{i,2},\ldots ,b_{i,D}\right)$. Meanwhile, the global best position, which can be understood as the best among all personal best positions, is stored in b such that $f\left(b\right)=\min_{i=1}^{N}f\left(b_{i}\right)$, where f(·) is the objective function to be minimized. In iteration *t*, the following equations will be applied to update the velocity as well as the position of each particle:

(24) $$\begin{array}{cc} v_{i}\left(t+1\right) & =\omega \left(t\right)v_{i}\left(t\right)+c_{1}\left(t\right)\xi_{1}\left[b_{i}\left(t\right)-x_{i}\left(t\right)\right]+c_{2}\left(t\right)\xi_{2}\left[b\left(t\right)-x_{i}\left(t\right)\right], \end{array}$$

(25) $$\begin{array}{cc} x_{i}\left(t+1\right) & =x_{i}\left(t\right)+v_{i}\left(t+1\right), \end{array}$$

where $\omega ,c_{1},c_{2}\geq 0$ are three key parameters of PSO, which are respectively referred to as the inertia weight, the cognitive acceleration coefficient and the social acceleration coefficient. Note that they can be set as time-variant parameters, which means their values may change with the iterations. $\xi_{1}$ and $\xi_{2}$ are random numbers generated from the uniform distribution U[0,1], which are introduced to provide randomness to the search process of PSO.

### 5.2. Encoding and Decoding of Solutions

We adopt a random key-based encoding scheme to represent the sequencing of cars in the paint shop as well as the allocation of cars to the buffer lanes. A potential solution to the problem (i.e., a particle) is expressed by a vector of *n* real numbers $x=(x_{1},x_{2},\ldots ,x_{n})$, where $x_{i}\in \left(0,L\right)$. The decimal part of $x_{i}$ reflects the relative order of car *i* in the processing sequence of the paint shop, while the integer part of $x_{i}$ specifies the assignment of a lane in the selectivity bank for car *i* when it leaves the paint shop.

In the decoding process, we apply the SPV (smallest position value) rule to determine the position for each car *i* in the processing sequence of paint shop ($k_{i}$). In particular, the *n* cars are sequenced according to a non-decreasing order of the corresponding values $\left\{x_{i}-\left\lfloor x_{i}\right\rfloor :i=1,\ldots ,n\right\}$. In addition, car *i* should enter the $l_{i}$-th lane of the selectivity bank after leaving the paint shop, where $l_{i}=\left\lceil x_{i}\right\rceil$.

An example with n=8 and L=3 is given in Table 2 to illustrate the decoding policy. Based on the given solution x=(1.80,2.19,0.21,1.32,0.95,2.05,1.54,0.82), the $k_{i}$ and $l_{i}$ values can be obtained as shown in the last row of the table. Clearly, the car sequence for the paint shop is achieved by sorting the decimal parts of $\left\{x_{1},\ldots ,x_{8}\right\}$ in a non-decreasing order, i.e., (6,2,3,4,7,1,8,5). After their completion in the paint shop, the 8 cars are to be moved into the 3-lane selectivity bank such that each lane is filled with the following subsequences: lane 1 with cars (3,8,5), lane 2 with cars (4,7,1), and lane 3 with cars (6,2).

### 5.3. Evaluation of Solutions

In the evaluation stage, the objective value TPE can be directly obtained based on the decoded processing sequence for paint shop. As for the TWT value, the B&B algorithm presented in Section 4 can be applied if we aim for an accurate evaluation of the TWT objective. However, the B&B algorithm is unavoidably time-consuming, and therefore we only use it for identification of global best solutions, when accuracy is important to distinguish between the solutions (detailed information will be given in Section 5.7). In the majority of time, it is advantageous to use a heuristic rule that provides a reasonably good approximation of the TWT value with a reasonable computational effort. For this purpose, we decide to adopt the dispatching rule named ATC (Apparent Tardiness Cost), which has been shown to be effective for minimizing the TWT criterion [35].

The ATC rule works in a simulation context. Every time when the machine becomes idle, a priority index is calculated for each eligible job (in the case of our problem, the first car currently parked in each lane), and the job with the highest index value will be selected to be processed next. The priority index $I_{j}\left(t\right)$ is a function of the time *t* at which the machine becomes idle and is defined for job *j* as follows (note that it has been adapted to our problem setting):

(26) $$I_{j}\left(t\right)=w_{j}\cdot \exp\left(-\frac{\max(d_{j}-1-t,0)}{K}\right),$$

where *K* is a scaling parameter for which we set a value of 4 (based on preliminary experiments). Meanwhile, *t* is represented by the number of jobs that have been finished at the time (due to the fact that $p_{j}=1$, ∀j).

### 5.4. Initialization of Solutions

Although purely random initialization is possible for the PSO algorithm to work, it is often better to start with a set of structured solutions so as to accelerate the convergence of optimization. We have designed the following procedures for generating such a group of initial solutions.

Step 1: Sort all the *n* cars in a non-decreasing order of their due dates. In the case of identical due dates, the cars with larger weights are prioritized. The sorted car sequence is denoted by *S*.
Step 2: Select a car randomly from the first τ positions of *S*, recorded as car *c*. Let F={c}, where *F* stands for the car sequence to be determined for the paint shop. Remove car *c* from *S*.
Step 3: Scan the first τ cars in the current *S*. Identify the car $c^{*}$ that leads to the minimum setup cost, i.e., such that $\delta_{\varepsilon \left(c\right)\varepsilon \left(c^{*}\right)}=\min_{c^{\prime }\in S_{\tau }}\left\{\delta_{\varepsilon \left(c\right)\varepsilon \left(c^{\prime }\right)}\right\}$, where ε(c) represents the color index of car *c* and $S_{\tau }$ denotes the subsequence which consists of the first τ cars in *S*.
Step 4: Append car $c^{*}$ to the end of sequence *F*. Remove car $c^{*}$ from *S*.
Step 5: If S≠∅, let $c=c^{*}$ and go back to Step 3. Otherwise, exit the procedure and output *F*.

The above procedure applies a window-based selection mechanism to determine the next car to be scheduled in an iterative manner. In each iteration, a car is selected from the first window of length τ in the initial sequence, which has been generated by the EDD (earliest due date) rule. The greedy selection policy together with the limitation imposed by the window length have contributed to achieving a trade-off between the pollution-minimization goal and the tardiness-reduction goal. The parameter τ is varied from 2 to n/2 so that a number of different sequences can be produced to diversify the initial solutions.

Note that an initial solution should also incorporate an allocation of cars to the buffer lanes. To find a suitable allocation associated with the painting sequence determined above, the following procedure is devised, which decides the lane for each car by considering the need for minimizing TWT in the downstream assembly shop.

Input: The processing sequence for paint shop, $F=\{c_{1},\ldots ,c_{n}\}$.
Step 1: Solve the single machine scheduling problem $1|p_{j}=1|\sum w_{j}T_{j}$ for the *n* cars. The optimal car sequence is recorded as $\pi^{*}$.
Step 2: For each car $c_{i}\in F$, find its position in $\pi^{*}$ and denote the position index by $\alpha (c_{i})$.
Step 3: Let i=1. Define ${\hat{\alpha }}_{l}=0$ for each lane l=1,…,L.
Step 4: For car $c_{i}$, if there exists *l* such that $\alpha \left(c_{i}\right)-{\hat{\alpha }}_{l}>0$, then let $l_{i}=\arg\min_{l\in \{1,\ldots ,L\}}\left\{\alpha \left(c_{i}\right)-{\hat{\alpha }}_{l}\right\}$; otherwise, let $l_{i}=\arg\max_{l\in \{1,\ldots ,L\}}\left\{\alpha \left(c_{i}\right)-{\hat{\alpha }}_{l}\right\}$.
Step 5: Push car $c_{i}$ to lane $l_{i}$. Let ${\hat{\alpha }}_{l_{i}}=\alpha \left(c_{i}\right)$.
Step 6: Let i←i+1. If i≤n, go back to Step 4. Otherwise, terminate the procedure.

Step 1 aims at the ideal processing sequence for paint shop (minimizing TWT with no precedence constraints). Then, the following steps are attempting to utilize the buffer system to transform the paint shop sequence *F* (which is already fixed) to a sequence that is as close to this ideal sequence as possible. When it is found that $\alpha \left(c_{i}\right)-{\hat{\alpha }}_{l}<0$ for all *l*, a violation against the ideal sequence occurs. It is generally impossible to reproduce the ideal sequence, so the focus is to use it as a guide for allocating the cars to the buffer lanes.

For example, we consider a case with 8 cars and a 3-lane buffer. Suppose that we have $F=\{c_{1},c_{2},\ldots ,c_{8}\}$ and $\pi^{*}=\left\{c_{4},c_{3},c_{7},c_{2},c_{6},c_{1},c_{8},c_{5}\right\}$. Therefore, it can be derived from Step 2 that $\left\{\alpha \left(c_{1}\right),\alpha \left(c_{2}\right),\ldots ,\alpha \left(c_{8}\right)\right\}=\left\{6,4,2,1,8,5,3,7\right\}$. Executing Steps 3–6 will lead to the following allocation of cars to the three lanes (expressed in the form “$c_{i}|\alpha \left(c_{i}\right)$”):

$$\begin{array}{cccc} \mathrm{Lane}\;1\text{:} & c_{1}|6 & c_{5}|8\\ \mathrm{Lane}\;2\text{:} & c_{2}|4 & c_{6}|5 & c_{8}|7\\ \mathrm{Lane}\;3\text{:} & c_{3}|2 & c_{4}|1 & c_{7}|3 \end{array}$$

which can produce the sequence $\pi^{\prime }=\left\{c_{3},c_{4},c_{7},c_{2},c_{6},c_{1},c_{8},c_{5}\right\}$ for the assembly shop. It is clear that $\pi^{\prime }$ differs from $\pi^{*}$ only in the first two positions. This violation is due to the step of inserting car $c_{4}|1$ immediately after car $c_{3}|2$ when $\alpha \left(c_{4}\right)-\alpha \left(c_{3}\right)<0$.

To transform an initialized schedule to the encoded form, we need to associate each car *i* with a real number $x_{i}$. Based on the initialization procedures, we assign $x_{c_{i}}=l_{i}-1+i/\left(n+1\right)$ for the car that is ranked at the *i*-th position in the sequence *F*. It is thereby ensured that the encoded solution $x=(x_{1},\ldots ,x_{n})$ can be converted back to the original schedule after it is decoded.

### 5.5. Time-Variant Parameters

To improve the search performance of the proposed MOPSO, we adopt time-variant settings for three major parameters, i.e., ω, $c_{1}$ and $c_{2}$.

The parameter ω determines the impact of the previous velocity on the current velocity of each particle. Setting a larger value for ω will promote extensive search for high-quality solutions, while setting a smaller value is beneficial for local search in the vicinity of the current position. It is well known that exploration and exploitation should be well balanced for any stochastic search algorithm to achieve good performance. The search pattern needs to be adjusted towards exploration in the beginning stage when there is limited information available about the landscape of search space. As the search progresses, more samples will be collected, and accordingly the search mode needs to be switched to more frequent exploitation so that the algorithm can make better use of the promising areas identified so far. Based on this logic, we apply a linearly decreasing policy for setting the parameter ω in iteration *t* (t=0,…,T), i.e.,

(27) $$\omega \left(t\right)=\left(\omega^{e}-\omega^{b}\right)\frac{t}{T}+\omega^{b},$$

where $\omega^{b}$ (resp. $\omega^{e}$) indicates the beginning value (resp. ending value) of the parameter (satisfying $\omega^{b}>\omega^{e}$), and *T* is the number of iterations for which ω is supposed to change over time (it is assumed that ω is fixed on the ending value from iteration T+1 onwards, if the algorithm is not terminated).

The acceleration coefficients, namely $c_{1}$ and $c_{2}$, can also produce a significant influence on the search behavior of PSO. Setting a larger value for $c_{1}$ and a smaller value for $c_{2}$ promotes distributed search, which leads to greater dispersion of particles in the search space. Conversely, setting a larger value for $c_{2}$ and a smaller value for $c_{1}$ will accelerate the convergence to the incumbent global best solution. Motivated by the fact, we apply a linearly decreasing policy for setting the parameter $c_{1}$ and a linearly increasing policy for setting the parameter $c_{2}$ in iteration *t* (t=0,…,T), i.e.,

(28) $$\begin{array}{cc} c_{1}\left(t\right) & =\left(c_{1}^{e}-c_{1}^{b}\right)\frac{t}{T}+c_{1}^{b}, \end{array}$$

(29) $$\begin{array}{cc} c_{2}\left(t\right) & =\left(c_{2}^{e}-c_{2}^{b}\right)\frac{t}{T}+c_{2}^{b}, \end{array}$$

where $c_{1}^{b}$ (resp. $c_{1}^{e}$) denotes the beginning value (resp. ending value) of parameter $c_{1}$ (satisfying $c_{1}^{b}>c_{1}^{e}$), and $c_{2}^{b}$ (resp. $c_{2}^{e}$) denotes the beginning value (resp. ending value) of parameter $c_{2}$ (satisfying $c_{2}^{b}<c_{2}^{e}$). The setting of *T* follows the same rule as stated above, and similarly, $c_{1}$ and $c_{2}$ will remain at their ending values if the algorithm continues after iteration *T*.

### 5.6. Sorting of Solutions

In multi-objective optimization context, Pareto dominance is the basic criterion for distinguishing the quality of solutions. Hence, when sorting a set of solutions denoted by X, we should prioritize the Pareto dominance relations by first dividing the set into *Q* subsets $X_{1},\ldots ,X_{Q}$ such that each solution $x\in X_{q}$ (2≤q≤Q) is dominated by at least one solution $x^{\prime }\in X_{q-1}$, and meanwhile, any pair of solutions from the same subset are mutually non-dominated. The algorithm for realizing such a Pareto-based sorting is detailed in [36].

The more important aspect of solution sorting lies in the technique for differentiating the solutions within each of these Pareto subsets (also called Pareto ranks), because the number of solutions in each $X_{q}$ (where there exists no mutual dominance relation) can be considerably large. The general idea for sorting the solutions in a non-dominated subset is to suppress the solutions that are crowded around by other solutions (regarding the objective space) and prioritize the solutions that are located in less crowded areas of the objective space. The motivation is to guarantee that the maintained solutions are well spread and can represent a wide variety of choices for the decision makers. The following procedure defines a crowding distance measure $u_{i}$ for each solution $x_{i}\in X_{q}$, which reflects the degrees of crowdedness in a quantitative manner.

Step 1: Evaluate the distance between each pair of solutions $x_{1},\;x_{2}\in X_{q}$ as $D\left(x_{1},x_{2}\right)={\left(\sum_{z=1}^{Z}{\left({\bar{d}}_{z}\left(x_{1},x_{2}\right)\right)}^{2}\right)}^{\frac{1}{2}}$, where ${\bar{d}}_{z}\left(x_{1},x_{2}\right)$ represents the normalized distance between $x_{1}$ and $x_{2}$ with respect to the *z*-th objective function, i.e., ${\bar{d}}_{z}\left(x_{1},x_{2}\right)=|f_{z}\left(x_{1}\right)-f_{z}\left(x_{2}\right)|/\left(f_{z}^{\max}-f_{z}^{\min}\right)$, with $f_{z}^{\max}$ (resp. $f_{z}^{\min}$) denoting the maximum (resp. minimum) value of the *z*-th objective in $X_{q}$. Z=2 refers to the number of objectives in our problem.
Step 2: For each solution $x_{i}\in X_{q}$, find the γ solutions that are situated most closely to $x_{i}$ in the objective space:
  (2.1) Let $D_{i}\left(1\right)=\min_{\theta }\left\{D\left(x_{i},x_{\theta }\right)|x_{\theta }\in X_{q}\backslash \left\{x_{i}\right\}\right\}$, $\theta \left(1\right)=\arg\min_{\theta }\left\{D\left(x_{i},x_{\theta }\right)|x_{\theta }\in X_{q}\backslash \left\{x_{i}\right\}\right\}$.
  (2.2) For g=2,…,γ, let $D_{i}\left(g\right)=\min_{\theta }\left\{D\left(x_{i},x_{\theta }\right)|x_{\theta }\in X_{q}\backslash \left\{\left\{x_{i}\right\}\cup \left\{x_{\theta (1)},\ldots ,x_{\theta (g-1)}\right\}\right\}\right\}$, $\theta \left(g\right)=\arg\min_{\theta }\left\{D\left(x_{i},x_{\theta }\right)|x_{\theta }\in X_{q}\backslash \left\{\left\{x_{i}\right\}\cup \left\{x_{\theta (1)},\ldots ,x_{\theta (g-1)}\right\}\right\}\right\}$.
Step 3: For each solution $x_{i}\in X_{q}$, calculate the crowding distance value as $u_{i}=\frac{1}{\gamma }\sum_{g=1}^{\gamma }D_{i}\left(g\right)$.

When evaluating $u_{i}$, γ is a parameter that should be set properly to balance accuracy and efficiency. It is suggested to set γ=4 for solving the problem studied in this paper. In a nutshell, we should sort solutions in a non-dominated set according to a decreasing order of their crowding distance values ($u_{i}$). Based on the sorting result, some solutions in the back rank may have to be abandoned during the evolutionary process due to the limited size of storage for elite solutions.

### 5.7. Handling of Personal Best and Global Best Solutions

In the proposed MOPSO algorithm, the mechanisms for identifying and updating personal best and global best solutions have been redesigned to suit the multi-objective optimization settings.

(1) Mechanism for handling personal best 

Based on the concept of Pareto optimality, the personal best that is maintained for each particle *i* should not be regarded as a single solution but rather a solution set which we denote by $B_{i}$. The personal best solution set $B_{i}$ is maintained according to the rules described as follows. Initially, it is assumed that $B_{i}=\left\{x_{i}\left(0\right)\right\}$. Then, in each iteration *t*, the following steps are performed after the newly obtained solution $x_{i}\left(t+1\right)$ has been evaluated. If $x_{i}\left(t+1\right)$ is found to be dominated by some existing solution in $B_{i}$, it will be neglected and $B_{i}$ is kept unchanged. If $x_{i}\left(t+1\right)$ is not dominated by any solution in $B_{i}$, it will be incorporated into $B_{i}$, and as a result, the originally existing solutions that are dominated by $x_{i}\left(t+1\right)$ will be eliminated from $B_{i}$ (if any).

To make sure that $B_{i}$ keeps the latest information acquired along the search trajectory of particle *i*, we set a common limit on the maximal size of $B_{i}$ and denote it by $m_{p}$. Whenever the actual size of $B_{i}$ reaches $m_{p}+1$, we simply remove the oldest solution in $B_{i}$, so that $B_{i}$ memorizes the most recent $m_{p}$ elite solutions that have been visited by particle *i*.

In the process of calculating the updated velocity of particle *i* by Equation (24), the required item $b_{i}$ is selected randomly from the personal best set $B_{i}$ following a uniform probability distribution, which means each candidate solution in $B_{i}$ is considered with equal probability $\mathrm{Pr}=1/|B_{i}|$.

(2) Mechanism for handling global best 

By definition, the global best is aimed at preserving the best-so-far solutions achieved by the entire swarm of particles. In the proposed MOPSO, the global best should also be characterized with a solution set, which we denote by *B*. Likewise, a limit of $m_{g}$ is placed on the maximal number of solutions that can be stored in *B*. In each iteration *t*, we apply the following procedure to update the global best solution set *B* based on the currently available personal best sets $B_{i}$ (i=1,…,N).

Step 1: Incorporate all solutions from $B_{1}\cup B_{2}\cup \cdots \cup B_{N}$ into *B*.
Step 2: Identify the first two Pareto ranks in *B* by performing a Pareto-based sorting of the solutions. Remove the solutions that belong to the other Pareto ranks from *B*.
Step 3: Evaluate each solution in *B* using the exact approach (i.e., getting the TWT objective value by the B&B method detailed in Section 4).
Step 4: Identify the first Pareto rank (i.e., the non-dominated solutions) in *B*. Remove the other solutions (those which are dominated) from *B*.
Step 5: Sort the solutions in *B* according to the crowding distance measure (c.f. Section 5.6).
Step 6: If $|B|>m_{g}$, remove from *B* the $\left(|B|-m_{g}\right)$ solutions that are ranked beyond the first $m_{g}$ places.

When updating particle *i*’s velocity with Equation (24), the item b is randomly selected from *B* based on the roulette wheel policy. Assuming that all solutions in *B* are sorted (as stated in Step 5), the probability of choosing the solution ranked at the *k*-th place is defined as:

(30) $$\mathrm{Pr}\left[k\right]=\frac{2(|B|+1-k)}{{\left|B\right|}^{2}+\left|B\right|},\;k=1,\ldots ,\left|B\right|.$$

Under such a probability assignment, the possibility of selecting each solution decreases linearly according to the sorted order. For example, if |B|=5, the selection probability assigned to each solution (in the sorted order) is 5/15, 4/15, 3/15, 2/15, 1/15, respectively.

### 5.8. Summary of the MOPSO Algorithm

**Figure 4:**
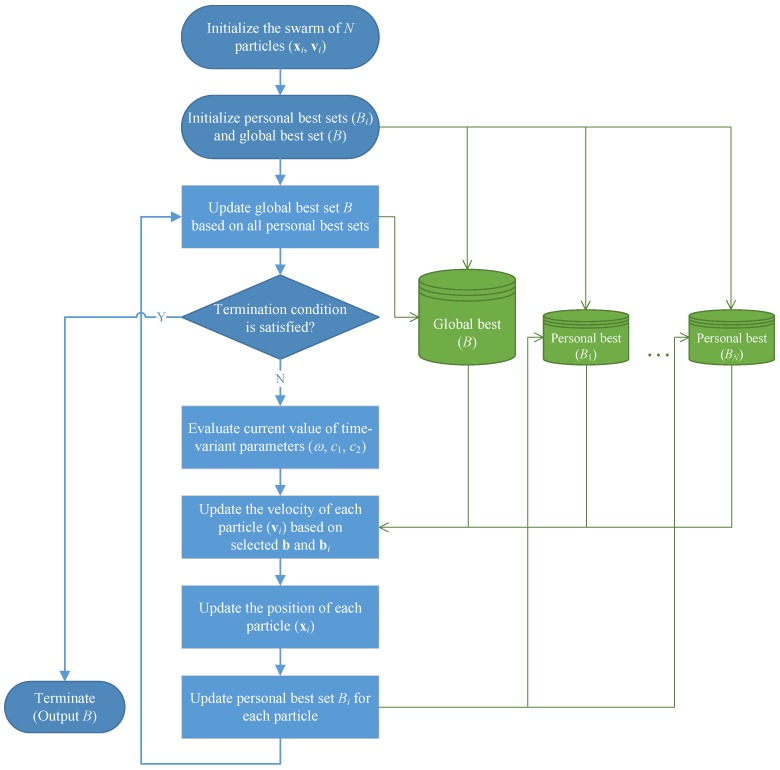
Flowchart of the proposed MOPSO algorithm.

We now provide an overall description of the proposed MOPSO algorithm. In addition, an associated flowchart is given as Figure 4 to help visualize the main structure of the algorithm (where the green lines indicate operations for storing information to and retrieving information from the personal best and global best solution sets).

Step 1: [Initialization] Apply the procedures given in Section 5.4 to generate the initial positions for a total of *N* particles, i.e., $\left\{x_{i}\left(0\right)|i=1,\ldots ,N\right\}$. Initialize the particles’ velocities by generating each component of $v_{i}\left(0\right)$ randomly from [−L/4,L/4]. Let $B_{i}=\left\{x_{i}\left(0\right)\right\}$ (for i=1,…,N) and B=∅. Define the iteration index t=0.
Step 2: [Global best] Update the global best solution set *B* based on the currently available personal best solution sets $B_{1},\ldots ,B_{N}$ by applying the procedure given in Section 5.7 (part (2)).
Step 3: [Termination test] If the termination condition is satisfied, terminate the algorithm with *B* as the output solutions. Otherwise, continue with the following steps.
Step 4: [Time-variant parameters] Evaluate the current value of the time-variant parameters, i.e., ω(t), $c_{1}\left(t\right)$ and $c_{2}\left(t\right)$, using Equations (27)–(29), respectively.
Step 5: [Velocity update] Determine $b_{i}$ by randomly selecting a solution from $B_{i}$. Determine b by applying the roulette wheel method to select a solution from *B*. Based on the selected $b_{i}$ and b, update the velocity for each particle *i* according to Equation (24), thus yielding $v_{i}\left(t+1\right)$.
Step 6: [Position update] Update the position for each particle *i* according to Equation (25), yielding $x_{i}\left(t+1\right)$. If any component of the new position vector falls below ε, it is reassigned with ε; if any component value exceeds L−ε, it is reassigned with L−ε (ε represents a very small constant, say, 0.001).
Step 7: [Personal best] Update the personal best solution set $B_{i}$ for each particle *i* with the newly obtained solution $x_{i}\left(t+1\right)$ by following the rules stated in Section 5.7 (part (1)).
Step 8: [Loop] Let t←t+1, and then return to Step 2.

## 6. Computational Experiments and Results

### 6.1. Experimental Setup

To examine the performance of the proposed MOPSO algorithm in solving the studied problem, an extensive set of test instances have been generated in the following specifications inspired by real-world production data.

- The number of cars (*n*) and the number of color options (*E*) are considered in a coordinated way at eight different levels, i.e., (n,E)∈{(50,3),(50,6),(100,6),(100,10),(150,9),(150,12),(200,10),(200,15)}.
- The number of lanes in the selectivity bank is considered at three levels, i.e., L∈{10,15,20}. We do not consider limitations on the capacity of each lane because it is assumed that the cars pass through the lanes dynamically.
- The required color for each car *i* is randomly determined from the set {1,…,E} with equal probability. The position-based due date of car *i* is set as $d_{i}=\zeta_{i}+1$, where $\zeta_{i}$ follows the binomial distribution B(n−1,0.5). The weight $w_{i}$ of car *i* is generated from the discrete uniform distribution U[1,10].
- The emission cost coefficient $\delta_{e_{1}e_{2}}$ is determined by $\mu \times (e_{2}-e_{1})$ for $e_{1}\leq e_{2}$, where μ is generated from the uniform distribution U[1,2]. Meanwhile, it is assumed that $\delta_{e_{2}e_{1}}=0.75\times \delta_{e_{1}e_{2}}$.

We have generated 8×3×5=120 test instances in accordance with the above standards (because we have 8 possible combinations of *n* and *E* as well as 3 possible values for *L*, and to increase reliability, 5 instances are generated for each scenario characterized by triplet (n,E,L)).

Following the standards for benchmarking multi-objective optimization algorithms, we adopt four performance indicators to assess the quality of obtained solutions. Suppose that X is a set of non-dominated solutions achieved by a certain optimization algorithm. Then, the performance indicators can be defined as follows.

- The ONVG (overall non-dominated vector generation) indicator [37] measures the number of solutions in the non-dominated solution set, i.e., $\mathrm{ONVG}\left(X\right)=\left|X\right|$. Higher ONVG values suggest that the corresponding algorithm is able to provide a wider range of choices for the ultimate decision-making.
- The CM (coverage metric) indicator [38] is defined on the basis of another non-dominated solution set (Y) for comparison with X. Formally, it is defined as
  (31) $$C\left(X,Y\right)=\frac{\left|\{y\in Y\;|\;\exists x\in X,\;x⪯y\}\right|}{\left|Y\right|},$$
  where x⪯y indicates the case that either x dominates y or f(x)=f(y) (i.e., having equal objective vectors). It is clear that C(X,Y) reflects the proportion of solutions in Y that are dominated by (or equal to) some solution in X. Therefore, a higher value of C(X,Y) suggests better performance of the algorithm which produces X.
- The $D_{\mathrm{av}}$ and $D_{\max}$ indicators [39] describe the distance between the solutions in X and a reference solution set R (ideally, the exact Pareto frontier of the problem) in the objective space. Formally, the distance metrics are defined as
  (32) $$\begin{array}{cc} D_{\mathrm{av}}\left(X,R\right) & =\frac{1}{\left|R\right|}\sum_{x^{\prime }\in R}\min_{x\in X}\left\{d\left(x,x^{\prime }\right)\right\}, \end{array}$$
  (33) $$\begin{array}{cc} D_{\max}\left(X,R\right) & =\max_{x^{\prime }\in R}\left\{\min_{x\in X}\left\{d\left(x,x^{\prime }\right)\right\}\right\}, \end{array}$$
  where the reference set R is usually composed of all non-dominated solutions obtained by the compared algorithms as an approximation to the real Pareto frontier if the latter is unknown. In the above equations, $d\left(x,x^{\prime }\right)=\max_{z=1}^{Z}\left\{\left(f_{z}\left(x\right)-f_{z}\left(x^{\prime }\right)\right)/\Delta_{z}\right\}$ where $\Delta_{z}=f_{z}^{\max}-f_{z}^{\min}$ is the value interval for the *z*-th objective function. Clearly, smaller values of $D_{\mathrm{av}}$ and $D_{\max}$ suggest that the solutions in X are closer to the estimated Pareto frontier.
- The TS (Tan’s Spacing) indicator [40] reflects how evenly the solutions in X are distributed. It is defined as
  (34) $$TS\left(X\right)=\frac{1}{\bar{D}}\sqrt{\frac{1}{\left|X\right|}\sum_{i=1}^{\left|X\right|}{\left(D_{i}-\bar{D}\right)}^{2}},$$
  where $\bar{D}=\left(1/\left|X\right|\right)\sum_{i=1}^{\left|X\right|}D_{i}$ with $D_{i}$ denoting the Euclidean distance between $x_{i}\in X$ and its closest neighbor solution in X (with regard to the objective space). Smaller values of TS indicate that the solutions are distributed more evenly and thus are more preferable for decision-making.

Based on preliminary experiments, the MOPSO parameter settings that will be adopted in the subsequent computational tests are listed as follows:

- Number of particles in the swarm: N=100;
- Beginning and ending values of inertia weight: $\omega^{b}=0.7$, $\omega^{e}=0.4$;
- Beginning and ending values of acceleration coefficients: $c_{1}^{b}=2.5$, $c_{1}^{e}=0.5$, $c_{2}^{b}=0.5$, $c_{2}^{e}=2.5$;
- Size limit on personal best solution sets: $m_{p}=4$;
- Size limit on global best solution set: $m_{g}=25$.

The beginning and ending values of parameters ω, $c_{1}$ and $c_{2}$ are chosen based on the suggestions given in [41]. The only difference lies in the beginning value of ω (i.e., $\omega^{b}$), which we have set to 0.7 in spite of the suggested value of 0.9. The main reason is that the proposed MOPSO algorithm relies on a heuristic initialization technique rather than completely randomized initial solutions, and consequently, our algorithm does not need a largely diversified search in the beginning stage. Preparatory experiments have shown that the setting of 0.7 for $\omega^{b}$ leads to the most desirable results.

### 6.2. Evaluation of Optimality

The proposed MOPSO is first compared with an exact MILP solver to reveal the ability of finding optimal solutions. The solutions for comparison have been obtained by the CPLEX solver based on weighted sum approach with objective function rewritten as f=λ·TPE+(1−λ)·TWT. The weighting coefficient λ is enumerated from 0.01 to 0.99 with a step size of 0.01. Considering that the exact solver is only able to address small-sized instances within reasonable time, the group of smallest test instances (i.e., with (n,E,L)=(50,3,10)) are utilized for making the comparison. The proposed MOPSO is executed 20 times independently for solving each instance (with 150 s allowed per run) and then the values of the four performance indicators are calculated. The resulting data are shown in the average sense in Table 3.

The results have clearly revealed that the proposed MOPSO has achieved remarkable solution quality. In the average sense, the solutions found by the MOPSO can cover 45% of the solutions obtained by CPLEX (with identical objective vectors). In addition, the MOPSO has realized low values of $D_{\mathrm{av}}$ and $D_{\max}$ (0.011 and 0.027, respectively), which suggests that the obtained solutions are sufficiently close to the CPLEX solutions. In terms of the ONVG metric, the MOPSO performs slightly worse than CPLEX (in view of the number of obtained solutions). However, when it comes to the TS metric, we can see that the evenness degree of solution distribution is comparable between the two approaches (1.39 vs. 1.37 on average). It is worth noting that the weighted sum method may fail to find certain solutions if the Pareto frontier is not fully convex. As a result, $C_{2}$ can be less than 1 for some instances. Finally, when computational time is taken into account, it is safe to conclude that the proposed MOPSO is much more efficient. By contrast, the CPLEX solver has consumed more than three hours for solving each instance.

### 6.3. Comparison with Typical MOEAs

To provide a systematic performance evaluation, we will compare the proposed MOPSO with high-performing multi-objective evolutionary algorithms (MOEAs) in the literature. In particular, the MOEA/D-ACO [42] and the pccsAMOPSO [43] have been selected for the comparison purpose. The former is an algorithm developed by combining the merits of the well-known MOEA/D (multi-objective evolutionary algorithm based on decomposition) and ACO (ant colony optimization), while the latter relies on a novel strategy called parallel cell coordinate system to balance convergence and diversity in the evolutionary process of multi-objective PSO. These two algorithms can represent the state-of-the-art techniques for solving generic multi-objective optimization problems. In the following computational experiments, the parameters of the two compared algorithms are determined based on the suggested values in the original publications and then fine-tuned to suit the features of our problem.

The proposed MOPSO as well as the two compared algorithms MOEA/D-ACO and pccsAMOPSO have been implemented with Visual C++ 2015 on a PC platform (Intel Core i7-4790 3.60GHz CPU, 16GB RAM, Windows 10 OS). To make sure that the comparisons are fair enough, we impose a hard limit on the computational time that is available for each algorithm in a single execution, i.e., $3\times n\times \frac{L}{10}$ seconds are allowed for solving an instance with *n* cars and *L* lanes in the selectivity bank. Under such settings, the tested algorithm must stop running and immediately output the current non-dominated solution set as soon as the time budget is used up. Consequently, the number of generations that can be evolved in the execution of an algorithm is not a fixed constant but dependent on the complexity of the tasks required to be performed in each iteration.

Each of the algorithms, including MOPSO, MOEA/D-ACO and pccsAMOPSO, has been run 20 times independently for solving each test instance. The averaged values of the four performance indicators resulting from the 20 runs are reported in Table 4, Table 5, Table 6, Table 7, Table 8, Table 9, Table 10 and Table 11 (grouped by *n*, the number of cars). In Table 8, Table 9, Table 10 and Table 11, X, Y and Z are respectively used to denote the solution set output by the MOPSO, MOEA/D-ACO and pccsAMOPSO. An asterisk in the tables denotes that the corresponding instance has the same size as the previous instance. To examine the statistical significance of the results, we have performed paired-sample *t*-tests on the indicator values in a pairwise manner (MOPSO vs. MOEA/D-ACO and MOPSO vs. pccsAMOPSO). The *p*-values obtained from the *t*-tests are reported in Table 12 and Table 13.

The following comments can be made regarding the computational results.

- Focusing on the ONVG indicator, we can see from the “Avg.” row that the proposed MOPSO has obtained more non-dominated solutions than the compared algorithms in the average sense (except in the comparison with MOEA/D-ACO on the group of 200-car instances). The statistical results also suggest that the differences are significant in most cases. The MOPSO outperforms the pccsAMOPSO consistently on all groups of instances, whereas the advantage over MOEA/D-ACO diminishes as the problem size grows. The relatively higher performance of MOEA/D-ACO on larger instances reveals the benefit of the decomposition-based optimization approach which promotes diversification and thereby handles huge solution spaces effectively. Overall, the number of obtained solutions increases with the problem size, which is not surprising because increased number of cars and buffer lanes will create more opportunities of making compromises between the emission objective and the tardiness objective.
- By taking a look at the $D_{\mathrm{av}}$ and $D_{\max}$ indicators, we find that the MOPSO has clearly outperformed both compared algorithms in terms of the average and maximum distances to the approximated Pareto frontier. In particular, the MOPSO has achieved the smallest average value of $D_{\mathrm{av}}$ among the three algorithms on 89 out of the 120 instances, and has achieved the smallest average value of $D_{\max}$ on 94 out of the 120 instances. The superior performance can be attributed to the enhanced search ability represented by the redesigned personal/global best handling mechanisms and some other problem-specific components of the algorithm. It is worthwhile to point out that the reference set R (used for calculating $D_{\mathrm{av}}$ and $D_{\max}$ in Equations (32) and ()), which represents the approximated Pareto frontier, is constructed by executing the two compared algorithms with sufficiently long iterations. This suggests that a bias has been created in favor of the MOEA/D-ACO and pccsAMOPSO. The remarkable performance of the proposed algorithm despite such an adverse condition is therefore quite convincing.
- By observing the TS indicator, we notice that the MOPSO has achieved the smallest average value among the three algorithms on 90 out of the 120 instances. The superior performance can be attributed to the improved solution sorting mechanism in the MOPSO which relies on a precisely defined crowding distance measure. In particular, our distance measure is based on the concept of Euclidean distances, which complies with the definition of the TS indicator. By contrast, the MOEA/D-ACO does not incorporate an explicit sorting mechanism based on crowdedness and therefore it results in the poorest performance in terms of the TS indicator. The pccsAMOPSO algorithm utilizes a novel distance measure called the parallel cell distance to estimate the density of solutions, which turns out to be effective in the process of solving our problem, especially for relatively smaller-sized instances. Overall, our distance measure has been proved to work more effectively under the TS indicator because of its accuracy for characterizing the distribution of solutions in the objective space. Finally, a noticeable trend is that the TS values generally increase with the size of instances. The degradation reflects the exponential explosion of solution spaces which adds to the difficulty of obtaining evenly-spaced non-dominated solutions.
- According to the CM indicator, it can be found that the average value of C(X,Y) stays above 0.90 and the average value of C(X,Z) stays above 0.94 across all the instances, which apparently means that a large portion of the solutions obtained by MOEA/D-ACO (Y) and pccsAMOPSO (Z) are dominated by or equal to (in terms of the objective vector) certain solutions output by the proposed algorithm (X). Meanwhile, the average values of C(Y,X) and C(Z,X) stay below 0.08 across all the instances, which indicates that the solution quality of the compared algorithms cannot match that of the proposed algorithm. To reveal the relative strengths of MOEA/D-ACO and pccsAMOPSO, we also report the average values of C(Y,Z) and C(Z,Y). The comparative results show that the MOEA/D-ACO maintains an advantage over the pccsAMOPSO, and the superiority becomes more apparent as the size of instances grows (noting that C(Y,Z) increases from 0.58 for n=50 to 0.71 for n=200 and C(Z,Y) decreases from 0.37 for n=50 to 0.25 for n=200), which validates high flexibility of the decomposition-based approach.
- As suggested by the statistical results given in Table 12 and Table 13 (one-tailed *p*-values), 29 of the 32 paired samples tested are significantly different in the statistical sense (under the significance level of 0.01). The 3 insignificant cases occur when we perform the test on the ONVG values. The underlying fact is that the number of non-dominated solutions obtained in each run of an algorithm is not very stable (it is more prone to random perturbations than other indicators). In general, the statistical results verify that the proposed MOPSO significantly outperforms the compared approaches for solving the bi-objective scheduling problem considered in this paper.

## 7. Conclusions

In this paper, we address an environment-aware production scheduling problem that arises in the car manufacturing industry. The problem has been defined as a bi-objective optimization model, in which one objective reflects the consideration of pollution-minimization requirements in the paint shop while the other objective characterizes the traditional goal of tardiness-minimization in the subsequent assembly shop. A mixed-integer linear programming formulation is developed to formally introduce the problem. Due to the high complexity and intractability, we devise a multi-objective particle swarm optimization algorithm (MOPSO) to solve the problem and obtain satisfactory production schedules within reasonable time. Since the MOPSO is aimed at optimizing the car sequence for the paint shop and the allocation of cars to the buffer lanes, the associated car sequencing decision for the assembly shop is treated as a separate subproblem. A branch-and-bound algorithm is proposed to solve this subproblem exactly, and also, a dispatching rule-based heuristic method is suggested to produce quick solutions for the subproblem. The former approach is utilized for identification of global best solutions in the MOPSO while the latter is used in all other situations when a solution needs to be evaluated. Such a design is inspired by the ordinal optimization philosophy (i.e., using crude models for solution evaluation will not lead to significant loss of optimal solutions) [44], which helps to bring down the computational burden of the whole algorithm.

In addition to the above considerations, the proposed MOPSO algorithm is characterized by the following important features:

- A random key-based encoding scheme which facilitates PSO implementation;
- A dedicated procedure for initialization of particles by exploiting problem-specific information;
- A set of time-variant parameters which help to achieve a better balance of extensive exploration and intensive exploitation by means of adjusting the search patterns dynamically;
- Some novel mechanisms to deal with multi-objective optimization (e.g., strategies for sorting solutions based on an accurately defined crowding distance measure and techniques for maintaining personal/global best solutions considering both Pareto dominance and diversity).

To test the proposed algorithm, we have conducted extensive computational experiments using 120 randomly generated instances of different sizes. In the optimality test, the MOPSO is able to produce high-quality solutions that are sufficiently close to the solutions obtained by the CPLEX solver for small-sized instances. In the principal experiments, the MOPSO is compared with two state-of-the-art multi-objective evolutionary algorithms under strictly the same computational time budget. The adopted performance indicators show that our algorithm has outperformed the compared approaches on a great majority of instances and in a statistically significant sense.

Future research will be focused on the following aspects. Firstly, it is interesting to incorporate the consideration of pollution-reduction requirements in other production units of automotive manufacturing systems, e.g., the body shop, which is responsible for building complete car bodies preceding the paint shop. Integrated scheduling of the entire production line will further contribute to reducing the overall pollutant emissions on the system level. Secondly, the optimization algorithm could be enhanced from several perspectives to ensure that it handles the integrated scheduling problem more efficiently. The central idea is to devise computationally fast local search components to be embedded into the MOPSO framework, especially the local search strategies that can make use of problem-specific structural properties (e.g., dominance property for a certain objective function).

## Figures and Tables

**Table 1:** Job data of the example instance.

Job Index (*j*)	1	2	3	4
Weight ($w_{j}$)	5	1	8	3
Due date ($d_{j}$)	2	2	1	1

**Table 2:** Illustration of the solution decoding scheme.

*i*	1	2	3	4	5	6	7	8
$x_{i}$	1.80	2.19	0.21	1.32	0.95	2.05	1.54	0.82
$\left(k_{i},l_{i}\right)$	(6,2)	(2,3)	(3,1)	(4,2)	(8,1)	(1,3)	(5,2)	(7,1)

**Table 3:** Comparison between MOPSO and CPLEX (with weighted sum method) based on the instances with (n,E,L)=(50,3,10).

No.	ONVG		$D_{\mathrm{av}}$		$D_{\max}$		TS		CM	
	MOPSO	CPLEX	MOPSO	CPLEX	MOPSO	CPLEX	MOPSO	CPLEX	$C_{1}$	$C_{2}$
1	9.86	12.00	0.009	0.000	0.018	0.000	1.44	1.33	0.40	1.00
2	12.73	14.00	0.011	0.002	0.028	0.012	1.32	1.37	0.43	0.98
3	10.46	12.00	0.013	0.000	0.030	0.000	1.30	1.44	0.47	1.00
4	10.91	13.00	0.008	0.000	0.024	0.000	1.42	1.40	0.39	1.00
5	12.68	14.00	0.016	0.004	0.034	0.010	1.49	1.30	0.54	0.98
Avg.	**11.33**	**13.00**	**0.011**	**0.001**	**0.027**	**0.004**	**1.39**	**1.37**	**0.45**	**0.99**

Note: $C_{1}=C\left(X,Y\right)$, $C_{2}=C\left(Y,X\right)$, where X (resp. Y) represents the solution set output by MOPSO (resp. CPLEX).

**Table 4:** Comparison of MOPSO with MOEA/D-ACO and pccsAMOPSO on the test instances with 50 cars.

No.	Size	MOPSO				MOEA/D-ACO				pccsAMOPSO			
		ONVG	$D_{\mathrm{av}}$	$D_{\max}$	TS	ONVG	$D_{\mathrm{av}}$	$D_{\max}$	TS	ONVG	$D_{\mathrm{av}}$	$D_{\max}$	TS
1	(50,3,10)	9.86	0.009	0.022	1.44	10.97	0.060	0.151	1.96	10.48	0.070	0.172	1.50
2	*	12.73	0.020	0.047	1.32	9.49	0.043	0.074	1.69	9.47	0.054	0.142	2.15
3	*	10.46	0.021	0.045	1.30	12.01	0.063	0.188	1.37	11.11	0.040	0.103	1.30
4	*	10.91	0.013	0.029	1.42	10.23	0.032	0.065	1.77	10.97	0.093	0.199	1.79
5	*	12.68	0.017	0.037	1.49	9.51	0.061	0.179	1.63	9.30	0.040	0.083	1.69
6	(50,3,15)	10.20	0.026	0.059	1.48	11.01	0.032	0.090	1.40	10.56	0.070	0.135	2.48
7	*	10.79	0.018	0.042	1.43	10.70	0.022	0.047	2.26	11.27	0.036	0.069	2.26
8	*	11.99	0.032	0.073	1.34	11.69	0.018	0.044	2.14	11.87	0.022	0.047	1.87
9	*	10.60	0.016	0.036	1.26	10.59	0.053	0.096	1.76	10.12	0.058	0.146	1.92
10	*	12.75	0.024	0.044	1.48	13.00	0.027	0.069	1.41	13.05	0.089	0.163	1.81
11	(50,3,20)	12.20	0.020	0.031	1.18	11.34	0.016	0.032	1.18	11.70	0.094	0.271	1.94
12	*	12.42	0.018	0.040	1.45	12.68	0.043	0.088	2.29	12.90	0.085	0.146	1.32
13	*	12.26	0.020	0.041	1.26	11.97	0.013	0.036	1.49	12.20	0.041	0.113	2.10
14	*	10.79	0.020	0.031	1.37	10.63	0.048	0.129	1.35	11.06	0.057	0.129	1.71
15	*	13.88	0.030	0.051	1.37	12.53	0.053	0.137	1.65	13.66	0.065	0.119	1.87
16	(50,6,10)	11.79	0.028	0.061	1.34	11.55	0.060	0.149	1.51	12.07	0.024	0.067	1.30
17	*	12.94	0.028	0.065	1.48	11.51	0.020	0.049	1.76	13.20	0.053	0.120	1.52
18	*	11.13	0.022	0.049	1.49	10.20	0.059	0.123	1.67	11.52	0.065	0.148	1.47
19	*	12.36	0.014	0.022	1.40	12.02	0.065	0.105	1.51	11.41	0.026	0.046	1.40
20	*	12.46	0.030	0.061	1.36	13.35	0.043	0.123	1.62	12.20	0.053	0.118	1.25
21	(50,6,15)	15.32	0.030	0.059	1.27	16.20	0.014	0.024	1.76	15.21	0.030	0.074	1.79
22	*	12.84	0.019	0.047	1.28	11.53	0.059	0.158	1.53	12.35	0.044	0.081	1.83
23	*	13.55	0.010	0.022	1.48	13.88	0.017	0.029	1.71	13.70	0.085	0.250	1.97
24	*	13.88	0.020	0.033	1.19	13.84	0.058	0.123	1.88	12.77	0.039	0.094	1.75
25	*	12.37	0.011	0.027	1.47	12.29	0.035	0.094	1.57	11.37	0.091	0.177	1.52
26	(50,6,20)	13.22	0.010	0.018	1.26	12.31	0.043	0.123	1.65	13.14	0.069	0.130	2.07
27	*	14.58	0.028	0.068	1.35	12.82	0.065	0.148	1.55	14.21	0.059	0.163	1.28
28	*	12.17	0.032	0.060	1.39	10.98	0.025	0.076	2.22	12.13	0.077	0.135	1.85
29	*	10.60	0.028	0.064	1.28	10.58	0.063	0.120	1.73	9.74	0.060	0.176	1.97
30	*	13.90	0.014	0.022	1.21	15.06	0.014	0.031	1.32	14.35	0.063	0.170	1.48
Avg.		**12.26**	**0.021**	**0.044**	**1.36**	**11.88**	**0.041**	**0.097**	**1.68**	**11.97**	**0.058**	**0.133**	**1.74**

**Table 5:** Comparison of MOPSO with MOEA/D-ACO and pccsAMOPSO on the test instances with 100 cars.

No.	Size	MOPSO				MOEA/D-ACO				pccsAMOPSO			
		ONVG	$D_{\mathrm{av}}$	$D_{\max}$	TS	ONVG	$D_{\mathrm{av}}$	$D_{\max}$	TS	ONVG	$D_{\mathrm{av}}$	$D_{\max}$	TS
31	(100,6,10)	16.75	0.037	0.075	1.43	15.48	0.062	0.169	1.64	16.01	0.131	0.265	1.52
32	*	16.72	0.048	0.107	1.42	17.62	0.034	0.057	1.81	13.85	0.023	0.044	1.87
33	*	15.38	0.038	0.093	1.35	15.62	0.044	0.085	1.63	14.01	0.123	0.328	1.60
34	*	16.53	0.044	0.085	1.36	14.98	0.026	0.046	1.34	15.20	0.095	0.183	1.34
35	*	15.66	0.026	0.056	1.36	14.57	0.073	0.169	1.54	15.28	0.033	0.095	1.49
36	(100,6,15)	15.97	0.047	0.098	1.49	15.59	0.067	0.109	2.19	12.91	0.076	0.203	1.90
37	*	17.02	0.027	0.061	1.26	18.50	0.083	0.171	1.25	16.19	0.044	0.080	1.34
38	*	16.25	0.035	0.079	1.30	14.65	0.079	0.210	1.21	14.68	0.094	0.240	1.29
39	*	14.86	0.037	0.065	1.69	14.54	0.074	0.215	2.51	12.06	0.057	0.136	1.72
40	*	17.60	0.036	0.078	1.55	16.61	0.059	0.166	1.45	15.23	0.061	0.151	1.49
41	(100,6,20)	17.48	0.049	0.109	1.52	15.81	0.053	0.123	1.65	16.49	0.023	0.059	1.57
42	*	18.98	0.045	0.076	1.37	17.82	0.067	0.147	2.04	17.86	0.115	0.292	1.37
43	*	17.14	0.041	0.076	1.66	16.01	0.052	0.102	1.52	16.64	0.062	0.126	1.92
44	*	17.06	0.027	0.064	1.53	17.88	0.079	0.196	1.50	14.32	0.129	0.228	1.59
45	*	15.76	0.033	0.053	1.44	14.29	0.073	0.151	1.68	13.92	0.071	0.181	1.62
46	(100,10,10)	16.82	0.036	0.088	1.35	16.77	0.030	0.085	1.56	14.41	0.144	0.368	1.65
47	*	16.63	0.029	0.045	1.70	17.42	0.067	0.123	1.83	13.33	0.085	0.159	1.92
48	*	17.70	0.023	0.037	1.30	17.55	0.027	0.080	1.46	16.12	0.089	0.234	1.45
49	*	18.59	0.035	0.087	1.42	19.18	0.047	0.090	1.97	16.79	0.030	0.081	1.47
50	*	20.80	0.039	0.083	1.56	21.43	0.030	0.056	2.24	16.99	0.061	0.175	1.75
51	(100,10,15)	19.05	0.035	0.055	1.40	19.00	0.063	0.170	1.31	18.37	0.076	0.163	1.87
52	*	18.39	0.041	0.071	1.72	16.96	0.042	0.086	1.97	17.77	0.099	0.200	1.97
53	*	17.86	0.041	0.074	1.59	17.49	0.072	0.141	1.73	16.61	0.140	0.317	1.58
54	*	17.44	0.026	0.052	1.53	15.79	0.050	0.130	1.58	15.82	0.024	0.066	1.80
55	*	16.38	0.039	0.062	1.70	14.61	0.024	0.068	2.08	14.97	0.110	0.200	1.86
56	(100,10,20)	15.76	0.020	0.031	1.36	17.05	0.068	0.117	1.34	13.99	0.034	0.080	1.52
57	*	21.07	0.021	0.033	1.39	22.25	0.061	0.131	1.92	19.70	0.118	0.213	1.50
58	*	20.31	0.050	0.116	1.27	21.47	0.084	0.190	1.32	16.37	0.055	0.131	1.52
59	*	16.49	0.046	0.099	1.72	15.38	0.078	0.173	1.71	14.12	0.115	0.310	1.84
60	*	22.24	0.043	0.090	1.64	23.25	0.049	0.132	2.34	18.44	0.124	0.224	1.83
Avg.		**17.49**	**0.036**	**0.073**	**1.48**	**17.19**	**0.057**	**0.130**	**1.71**	**15.61**	**0.081**	**0.184**	**1.64**

**Table 6:** Comparison of MOPSO with MOEA/D-ACO and pccsAMOPSO on the test instances with 150 cars.

No.	Size	MOPSO				MOEA/D-ACO				pccsAMOPSO			
		ONVG	$D_{\mathrm{av}}$	$D_{\max}$	TS	ONVG	$D_{\mathrm{av}}$	$D_{\max}$	TS	ONVG	$D_{\mathrm{av}}$	$D_{\max}$	TS
61	(150,9,10)	18.04	0.061	0.150	1.41	16.51	0.060	0.156	1.72	15.54	0.118	0.216	1.47
62	*	18.42	0.056	0.086	1.42	18.79	0.095	0.259	1.46	16.62	0.118	0.260	1.59
63	*	19.80	0.048	0.094	1.67	17.93	0.118	0.305	2.21	16.49	0.065	0.182	1.67
64	*	17.39	0.023	0.046	1.49	18.01	0.033	0.087	1.56	14.70	0.029	0.079	1.61
65	*	17.15	0.066	0.118	1.58	16.63	0.093	0.186	1.84	14.80	0.125	0.232	1.68
66	(150,9,15)	21.60	0.036	0.087	1.37	22.49	0.037	0.072	1.93	19.87	0.044	0.102	1.96
67	*	20.53	0.023	0.039	1.42	21.35	0.130	0.377	1.66	17.88	0.173	0.404	1.54
68	*	17.23	0.064	0.154	1.51	16.10	0.141	0.269	2.26	14.39	0.107	0.298	1.55
69	*	21.43	0.048	0.080	1.59	19.34	0.136	0.225	2.58	19.69	0.107	0.254	2.06
70	*	19.09	0.053	0.100	1.63	19.85	0.078	0.172	2.37	17.32	0.062	0.114	1.81
71	(150,9,20)	21.41	0.020	0.041	1.44	21.22	0.121	0.292	1.57	19.13	0.078	0.136	2.14
72	*	23.23	0.055	0.132	1.42	21.64	0.065	0.195	1.94	20.67	0.069	0.131	2.12
73	*	18.09	0.028	0.050	1.50	17.24	0.075	0.182	2.17	16.11	0.170	0.400	1.65
74	*	23.27	0.044	0.085	1.59	23.70	0.046	0.095	2.00	19.93	0.151	0.326	2.07
75	*	19.21	0.028	0.049	1.65	18.97	0.135	0.248	2.47	17.13	0.140	0.251	2.43
76	(150,12,10)	19.57	0.055	0.111	1.57	18.54	0.042	0.087	1.78	16.72	0.138	0.246	1.50
77	*	22.06	0.028	0.051	1.64	21.79	0.054	0.106	2.56	17.95	0.051	0.129	1.77
78	*	22.10	0.045	0.078	1.55	20.80	0.053	0.147	1.96	19.87	0.035	0.081	1.92
79	*	23.50	0.019	0.040	1.35	22.75	0.159	0.465	1.61	21.01	0.112	0.193	1.92
80	*	18.64	0.019	0.043	1.49	19.26	0.033	0.098	1.81	16.95	0.151	0.314	2.26
81	(150,12,15)	20.83	0.061	0.093	1.60	19.48	0.034	0.094	1.76	18.61	0.105	0.295	1.60
82	*	20.19	0.040	0.097	1.66	20.78	0.085	0.211	2.63	18.12	0.080	0.171	2.34
83	*	21.87	0.049	0.113	1.50	22.24	0.044	0.086	1.85	18.74	0.102	0.257	1.87
84	*	22.71	0.044	0.072	1.53	21.92	0.123	0.260	2.22	20.51	0.105	0.226	2.04
85	*	22.78	0.032	0.073	1.38	22.67	0.092	0.227	2.20	20.03	0.065	0.138	1.54
86	(150,12,20)	26.75	0.040	0.092	1.40	26.98	0.119	0.303	1.38	22.77	0.086	0.211	1.66
87	*	21.40	0.065	0.147	1.50	21.74	0.098	0.219	2.20	19.36	0.160	0.434	1.79
88	*	21.60	0.025	0.046	1.67	20.85	0.119	0.265	2.07	17.76	0.103	0.193	1.87
89	*	21.15	0.060	0.104	1.38	20.21	0.033	0.086	1.65	18.92	0.051	0.126	1.71
90	*	23.54	0.041	0.099	1.70	22.74	0.161	0.424	2.25	21.61	0.111	0.286	2.22
Avg.		**20.82**	**0.043**	**0.086**	**1.52**	**20.42**	**0.087**	**0.207**	**1.99**	**18.31**	**0.100**	**0.223**	**1.85**

**Table 7:** Comparison of MOPSO with MOEA/D-ACO and pccsAMOPSO on the test instances with 200 cars.

No.	Size	MOPSO				MOEA/D-ACO				pccsAMOPSO			
		ONVG	$D_{\mathrm{av}}$	$D_{\max}$	TS	ONVG	$D_{\mathrm{av}}$	$D_{\max}$	TS	ONVG	$D_{\mathrm{av}}$	$D_{\max}$	TS
91	(200,10,10)	22.75	0.044	0.100	1.52	23.92	0.028	0.067	1.57	20.82	0.117	0.237	1.71
92	*	18.64	0.091	0.213	1.44	18.88	0.044	0.127	1.61	17.44	0.124	0.219	1.64
93	*	19.81	0.058	0.125	1.44	20.16	0.161	0.436	1.75	17.97	0.188	0.549	1.56
94	*	19.02	0.085	0.155	1.68	18.80	0.149	0.380	1.83	17.44	0.227	0.396	1.81
95	*	18.64	0.025	0.043	1.67	19.60	0.088	0.228	2.27	16.64	0.161	0.296	2.03
96	(200,10,15)	19.24	0.024	0.043	1.60	20.35	0.055	0.116	2.50	18.31	0.032	0.090	2.01
97	*	21.94	0.078	0.142	1.39	23.15	0.039	0.102	1.85	20.54	0.103	0.218	1.67
98	*	21.33	0.074	0.116	1.53	20.28	0.092	0.159	2.05	20.56	0.070	0.152	1.69
99	*	22.71	0.094	0.167	1.70	21.36	0.037	0.089	1.92	21.36	0.121	0.264	2.32
100	*	21.79	0.079	0.124	1.78	22.28	0.114	0.325	2.55	19.48	0.023	0.068	1.71
101	(200,10,20)	21.58	0.054	0.087	1.67	21.54	0.058	0.145	2.11	18.75	0.056	0.133	1.91
102	*	24.34	0.051	0.111	1.49	26.85	0.159	0.436	2.36	20.58	0.133	0.385	1.65
103	*	22.38	0.090	0.177	1.78	22.15	0.219	0.365	2.07	20.92	0.211	0.491	2.36
104	*	20.74	0.065	0.160	1.68	22.11	0.163	0.340	1.88	20.05	0.157	0.303	1.82
105	*	22.51	0.038	0.065	1.87	21.06	0.142	0.322	2.13	20.51	0.131	0.288	1.92
106	(200,15,10)	27.13	0.087	0.172	1.65	26.58	0.103	0.205	2.59	23.34	0.141	0.249	1.73
107	*	26.67	0.066	0.120	1.66	28.61	0.052	0.091	2.69	24.31	0.207	0.421	1.67
108	*	22.95	0.037	0.089	1.70	24.09	0.183	0.516	1.92	19.77	0.041	0.108	2.20
109	*	21.41	0.025	0.062	1.50	19.99	0.209	0.606	1.78	18.56	0.162	0.351	2.10
110	*	24.86	0.095	0.210	1.72	23.40	0.190	0.520	2.46	24.05	0.121	0.244	1.61
111	(200,15,15)	21.15	0.094	0.230	1.84	21.81	0.110	0.310	2.65	17.86	0.097	0.254	1.82
112	*	22.02	0.062	0.101	1.91	22.34	0.083	0.244	2.34	20.89	0.222	0.513	1.88
113	*	28.67	0.078	0.158	1.41	30.25	0.088	0.145	1.88	25.61	0.114	0.316	1.43
114	*	20.17	0.082	0.170	1.40	21.50	0.145	0.421	1.52	17.83	0.203	0.515	1.39
115	*	24.16	0.055	0.097	1.77	25.85	0.097	0.172	2.17	20.58	0.164	0.476	2.00
116	(200,15,20)	26.08	0.062	0.148	1.56	27.06	0.043	0.115	2.34	24.23	0.128	0.348	1.95
117	*	23.03	0.085	0.185	1.72	23.67	0.082	0.164	2.33	22.10	0.056	0.112	1.69
118	*	23.17	0.075	0.122	1.40	23.46	0.218	0.545	1.72	20.24	0.174	0.422	1.84
119	*	27.55	0.032	0.060	1.51	29.95	0.131	0.362	1.99	26.51	0.112	0.321	1.86
120	*	24.76	0.089	0.172	1.84	25.14	0.170	0.460	2.84	21.97	0.095	0.166	1.97
Avg.		**22.71**	**0.066**	**0.131**	**1.63**	**23.21**	**0.115**	**0.284**	**2.12**	**20.64**	**0.130**	**0.297**	**1.83**

**Table 8:** Coverage metrics for comparing the algorithms on the test instances with 50 cars.

No.	Size	C(X,Y)	C(Y,X)	C(X,Z)	C(Z,X)	C(Y,Z)	C(Z,Y)
1	(50,3,10)	1.00	0.00	0.88	0.10	0.93	0.01
2	*	0.91	0.09	0.95	0.04	0.52	0.43
3	*	0.86	0.12	0.98	0.00	0.41	0.59
4	*	0.93	0.05	0.88	0.10	0.44	0.55
5	*	0.87	0.10	0.87	0.11	0.37	0.57
6	(50,3,15)	0.87	0.11	1.00	0.00	0.68	0.26
7	*	0.99	0.00	0.94	0.04	0.86	0.11
8	*	0.93	0.04	0.87	0.11	0.62	0.36
9	*	0.85	0.14	1.00	0.00	0.62	0.33
10	*	0.87	0.09	0.96	0.01	0.42	0.54
11	(50,3,20)	0.88	0.06	0.88	0.10	0.39	0.53
12	*	0.85	0.13	0.88	0.09	0.71	0.21
13	*	0.93	0.03	1.00	0.00	0.39	0.58
14	*	0.97	0.02	0.89	0.07	0.41	0.57
15	*	0.98	0.01	0.95	0.02	0.40	0.59
16	(50,6,10)	0.97	0.00	0.93	0.07	0.59	0.36
17	*	1.00	0.00	1.00	0.00	0.89	0.05
18	*	0.91	0.09	0.91	0.06	0.56	0.42
19	*	0.87	0.09	0.85	0.15	0.43	0.50
20	*	1.00	0.00	0.89	0.06	1.00	0.00
21	(50,6,15)	0.97	0.01	0.89	0.08	0.60	0.38
22	*	0.86	0.12	1.00	0.00	0.49	0.46
23	*	0.90	0.06	0.84	0.15	0.55	0.38
24	*	0.91	0.06	0.99	0.00	0.61	0.33
25	*	0.91	0.02	1.00	0.00	0.41	0.51
26	(50,6,20)	0.86	0.11	0.91	0.04	0.97	0.00
27	*	0.82	0.18	0.98	0.00	0.69	0.24
28	*	0.94	0.04	0.98	0.00	0.60	0.32
29	*	0.81	0.15	0.85	0.13	0.54	0.38
30	*	0.88	0.11	0.95	0.00	0.42	0.56
Avg.		**0.91**	**0.07**	**0.93**	**0.05**	**0.58**	**0.37**

**Table 9:** Coverage metrics for comparing the algorithms on the test instances with 100 cars.

No.	Size	C(X,Y)	C(Y,X)	C(X,Z)	C(Z,X)	C(Y,Z)	C(Z,Y)
31	(100,6,10)	0.87	0.12	0.92	0.03	0.70	0.23
32	*	0.81	0.14	1.00	0.00	0.65	0.34
33	*	0.82	0.15	1.00	0.00	0.53	0.46
34	*	0.85	0.10	0.94	0.04	0.76	0.21
35	*	0.99	0.00	1.00	0.00	0.78	0.16
36	(100,6,15)	0.98	0.00	0.89	0.10	0.78	0.21
37	*	0.90	0.08	1.00	0.00	0.93	0.00
38	*	0.99	0.00	0.84	0.11	0.44	0.49
39	*	0.96	0.00	0.96	0.01	0.78	0.15
40	*	0.98	0.00	0.92	0.08	0.44	0.48
41	(100,6,20)	0.90	0.09	1.00	0.00	0.39	0.58
42	*	0.82	0.13	0.86	0.09	0.78	0.16
43	*	0.82	0.13	0.87	0.09	0.38	0.54
44	*	0.92	0.06	0.92	0.03	0.57	0.43
45	*	0.98	0.01	0.87	0.09	0.65	0.31
46	(100,10,10)	1.00	0.00	0.86	0.11	0.81	0.15
47	*	0.99	0.00	0.91	0.04	0.41	0.54
48	*	0.84	0.15	0.90	0.05	0.42	0.52
49	*	0.98	0.01	0.95	0.01	0.60	0.32
50	*	0.88	0.07	0.90	0.07	0.73	0.21
51	(100,10,15)	0.93	0.04	0.96	0.00	0.90	0.05
52	*	0.88	0.10	1.00	0.00	0.92	0.05
53	*	0.95	0.00	1.00	0.00	0.52	0.42
54	*	1.00	0.00	0.88	0.12	0.97	0.03
55	*	0.91	0.02	0.95	0.04	0.94	0.02
56	(100,10,20)	1.00	0.00	0.93	0.04	0.47	0.46
57	*	0.83	0.13	0.98	0.00	0.69	0.26
58	*	0.88	0.11	0.96	0.02	0.43	0.50
59	*	1.00	0.00	0.99	0.01	0.61	0.35
60	*	0.98	0.01	1.00	0.00	0.60	0.39
Avg.		**0.92**	**0.05**	**0.94**	**0.04**	**0.65**	**0.30**

**Table 10:** Coverage metrics for comparing the algorithms on the test instances with 150 cars.

No.	Size	C(X,Y)	C(Y,X)	C(X,Z)	C(Z,X)	C(Y,Z)	C(Z,Y)
61	(150,9,10)	0.83	0.12	1.00	0.00	0.55	0.37
62	*	0.97	0.00	1.00	0.00	0.91	0.08
63	*	0.92	0.04	1.00	0.00	0.94	0.02
64	*	0.97	0.02	0.95	0.01	0.37	0.61
65	*	1.00	0.00	0.88	0.08	0.81	0.13
66	(150,9,15)	0.83	0.14	0.93	0.04	0.74	0.19
67	*	0.88	0.11	1.00	0.00	0.37	0.61
68	*	0.95	0.04	1.00	0.00	0.81	0.18
69	*	0.86	0.12	0.88	0.07	0.57	0.42
70	*	0.94	0.01	1.00	0.00	0.55	0.38
71	(150,9,20)	0.84	0.16	0.92	0.05	0.65	0.29
72	*	0.90	0.08	0.93	0.05	0.39	0.61
73	*	0.84	0.11	0.90	0.10	0.47	0.49
74	*	1.00	0.00	0.92	0.03	0.96	0.00
75	*	0.96	0.01	0.86	0.12	0.74	0.20
76	(150,12,10)	0.99	0.00	0.95	0.00	0.41	0.56
77	*	1.00	0.00	0.98	0.00	0.61	0.31
78	*	0.96	0.00	1.00	0.00	0.57	0.43
79	*	0.82	0.16	0.93	0.06	0.79	0.14
80	*	0.91	0.07	0.96	0.02	1.00	0.00
81	(150,12,15)	0.81	0.18	0.85	0.12	0.93	0.00
82	*	0.87	0.12	1.00	0.00	0.39	0.52
83	*	0.83	0.14	0.88	0.09	0.62	0.37
84	*	0.91	0.08	0.93	0.02	0.73	0.22
85	*	0.82	0.16	0.96	0.01	0.73	0.22
86	(150,12,20)	0.89	0.10	0.89	0.05	0.78	0.15
87	*	0.91	0.05	0.90	0.06	0.40	0.52
88	*	0.86	0.10	0.92	0.03	0.44	0.53
89	*	0.87	0.08	1.00	0.00	0.79	0.17
90	*	0.86	0.08	0.96	0.01	0.83	0.15
Avg.		**0.90**	**0.08**	**0.94**	**0.03**	**0.66**	**0.30**

**Table 11:** Coverage metrics for comparing the algorithms on the test instances with 200 cars.

No.	Size	C(X,Y)	C(Y,X)	C(X,Z)	C(Z,X)	C(Y,Z)	C(Z,Y)
91	(200,10,10)	0.85	0.11	1.00	0.00	0.68	0.29
92	*	0.96	0.01	0.93	0.03	0.85	0.14
93	*	0.81	0.17	1.00	0.00	0.65	0.35
94	*	0.84	0.15	1.00	0.00	0.89	0.11
95	*	0.96	0.00	0.93	0.03	0.73	0.21
96	(200,10,15)	0.88	0.11	1.00	0.00	0.72	0.23
97	*	0.96	0.00	0.87	0.12	0.89	0.04
98	*	0.82	0.15	0.94	0.02	0.41	0.51
99	*	0.81	0.15	1.00	0.00	0.67	0.29
100	*	0.85	0.11	0.89	0.06	0.84	0.13
101	(200,10,20)	0.86	0.13	0.92	0.07	0.96	0.00
102	*	0.98	0.00	1.00	0.00	0.55	0.42
103	*	1.00	0.00	1.00	0.00	0.99	0.00
104	*	0.93	0.05	0.92	0.04	0.86	0.06
105	*	0.98	0.01	0.86	0.09	0.91	0.02
106	(200,15,10)	0.94	0.03	1.00	0.00	0.39	0.56
107	*	0.96	0.00	1.00	0.00	0.98	0.00
108	*	0.97	0.00	0.87	0.12	0.88	0.07
109	*	0.95	0.02	1.00	0.00	0.39	0.59
110	*	0.86	0.12	0.95	0.02	0.80	0.12
111	(200,15,15)	0.91	0.06	1.00	0.00	0.49	0.49
112	*	0.82	0.15	0.85	0.13	0.40	0.54
113	*	0.92	0.02	0.87	0.13	0.98	0.00
114	*	0.97	0.00	0.99	0.00	0.64	0.28
115	*	0.84	0.12	1.00	0.00	0.92	0.06
116	(200,15,20)	0.84	0.11	0.92	0.04	0.42	0.55
117	*	0.86	0.12	0.97	0.00	0.90	0.07
118	*	0.81	0.15	0.89	0.11	0.45	0.48
119	*	0.87	0.13	1.00	0.00	0.53	0.42
120	*	0.81	0.13	0.90	0.06	0.56	0.38
Avg.		**0.89**	**0.08**	**0.95**	**0.04**	**0.71**	**0.25**

**Table 12:** The *p*-values resulting from the *t*-tests for MOPSO and MOEA/D-ACO.

*n*	ONVG	$D_{\mathrm{av}}$	$D_{\max}$	TS
50	$4.14\times 10^{-2}$	$4.06\times 10^{-6}$	$1.16\times 10^{-6}$	$2.90\times 10^{-7}$
100	$6.66\times 10^{-2}$	$4.47\times 10^{-6}$	$1.87\times 10^{-6}$	$4.77\times 10^{-5}$
150	$8.49\times 10^{-3}$	$5.85\times 10^{-6}$	$1.74\times 10^{-6}$	$4.30\times 10^{-10}$
200	$9.87\times 10^{-3}$	$8.14\times 10^{-5}$	$1.28\times 10^{-5}$	$1.40\times 10^{-10}$

**Table 13:** The *p*-values resulting from the *t*-tests for MOPSO and pccsAMOPSO.

*n*	ONVG	$D_{\mathrm{av}}$	$D_{\max}$	TS
50	$5.74\times 10^{-2}$	$1.64\times 10^{-9}$	$3.20\times 10^{-9}$	$5.99\times 10^{-7}$
100	$3.93\times 10^{-11}$	$2.89\times 10^{-7}$	$5.79\times 10^{-8}$	$2.88\times 10^{-7}$
150	$4.19\times 10^{-19}$	$1.91\times 10^{-8}$	$8.94\times 10^{-9}$	$5.48\times 10^{-8}$
200	$6.59\times 10^{-13}$	$8.72\times 10^{-7}$	$3.58\times 10^{-7}$	$4.49\times 10^{-6}$

## Notations

The following notations are used in this paper:

*n*	number of cars
*E*	number of color options
*L*	number of lanes in the buffer
$d_{i}$	position-based due date of car *i*
$w_{i}$	priority weight of car *i*
$T_{i}$	tardiness of car *i* in the assembly shop
$\delta_{e_{1}e_{2}}$	amount of emission incurred when switching from color $e_{1}$ to color $e_{2}$
$\beta_{ei}$	a 0−1 constant which equals 1 iff car *i* requires color *e*
TPE	total pollutant emissions in the paint shop
TWT	total weighted tardiness in the assembly shop
LB	lower bound of TWT used by the branch-and-bound algorithm
$x_{i}$	position of particle *i* in the MOPSO
$v_{i}$	velocity of particle *i* in the MOPSO
*t*	iteration index in the MOPSO
ω(t)	time-variant inertia weight in the MOPSO
$c_{1}\left(t\right)$, $c_{2}\left(t\right)$	time-variant acceleration coefficients in the MOPSO
$b_{i}$	personal best solution (associated with particle *i*) selected for position update
b	global best solution selected for position update
$B_{i}$	personal best solution set maintained for particle *i*
*B*	global best solution set
$m_{p}$	maximum size of personal best solution sets
$m_{g}$	maximum size of global best solution set
*N*	number of particles in the MOPSO
$u_{i}$	crowding distance value for solution *i* in a non-dominated set
γ	truncation parameter in the calculation of $u_{i}$
ONVG	number of non-dominated solutions in the obtained solution set
CM	coverage metric which reflects the dominance relation between two solution sets
$D_{\mathrm{av}}$, $D_{\max}$	distance metrics which depict how far the solutions are situated from the true Pareto frontier
TS	spacing metric which describes how evenly the solutions in a set are distributed
